# Systematic review of the impact of intestinal microbiota on vaccine responses

**DOI:** 10.1038/s41541-024-01000-0

**Published:** 2024-12-20

**Authors:** Cristina Ardura-Garcia, Nigel Curtis, Petra Zimmermann

**Affiliations:** 1 Department of Paediatrics, Fribourg Hospital, Fribourg, Switzerland; 2 Cambodia Oxford Medical Research Unit, Angkor Hospital for Children, Siem Reap, Cambodia; 3 Department of Paediatrics, The University of Melbourne, Parkville, VIC Australia; 4 Infectious Diseases Research Group, Murdoch Children’s Research Institute, Parkville, VIC Australia; 5 Infectious Diseases Unit, The Royal Children’s Hospital Melbourne, Parkville, VIC Australia; 6 Department for Community Health, Faculty of Science and Medicine, University of Fribourg, Fribourg, Switzerland

**Keywords:** Medical research, Health care

## Abstract

The intestinal microbiota plays a critical role in host immunity and might contribute to the significant variation between individuals’ vaccine responses. A systematic search was done using MEDLINE and Embase to identify original human studies investigating the association between intestinal microbiota composition and humoral and cellular vaccine responses. In total, 30 publications (26 studies, 14 in infants, 12 in adults), were included. Of these, 26 publications found an association between intestinal microbiota composition and vaccine responses. A beneficial effect of Actynomycetota (particularly *Bifidobacterium*) and a detrimental effect of Pseudomonadota (particularly Gammaproteobacteria) were observed across studies. Study designs were highly heterogenous, with variation in vaccine type, outcome measure, timing of stool analysis and analysis methods. Overall, studies support the concept that the composition of the intestinal microbiota influences vaccine responses. Further adequately powered studies are needed to confirm this association and inform potential microbiota-targeted interventions to optimise vaccine responses.

## Introduction

Vaccines save ~2–3 million lives per year^1^. Despite their effectiveness, vaccine responses are highly variable between individuals^2,3^. This has been documented for many types of vaccines, including conjugated pneumococcal (PCV), hepatitis B (HepB), inactivated influenza (IIV) and Bacillus Calmette–Guérin (BCG) vaccines^4–6^. Factors contributing to this variability include age (lower responses in infants and elderly)^7–9^, geographic location (lower responses in low-middle income countries (LMICs) compared with high-income countries (HICs))^9–13^ and comorbidities^9^. Variability in vaccine responses has been observed after both oral and parental vaccines and encompasses both humoral and cellular responses^14^. Understanding host factors that influence vaccine responses is key to implementing strategies to increase vaccine efficacy.

The intestinal microbiota has emerged as a key host factor that may modulate vaccine responses^14,15^. As with vaccine responses, the composition of the intestinal microbiota also varies with age, geographic location and comorbidities^3^. The intestinal microbiota is less stable and diverse during infancy and old age compared with late childhood and adulthood^16^. Several studies have reported important differences between those in LMICs and HICs in the diversity of intestinal microbiota and relative abundance of specific bacteria^17–20^.

In a systematic review in 2018, only four studies were identified that assessed the relationship between the composition of intestinal microbiota and vaccine responses. Despite the sparsity of studies, a higher relative abundance of Actynomycetota (previously known as Actinobacteria) was consistently associated with higher vaccine responses and a higher relative abundance of Bacteroidota (previous Bacteroidetes) with lower responses, while the association between the relative abundance of the phyla Bacillota (previously Firmicutes) and Pseudomonadota (previous Proteobacteria) and vaccine responses varied for different genera and species^15^. As there have now been many more studies on this topic, we present here an updated review of studies that investigated the association between the intestinal microbiota and both humoral and cellular vaccine responses.

## Results

**Fig. 1:**
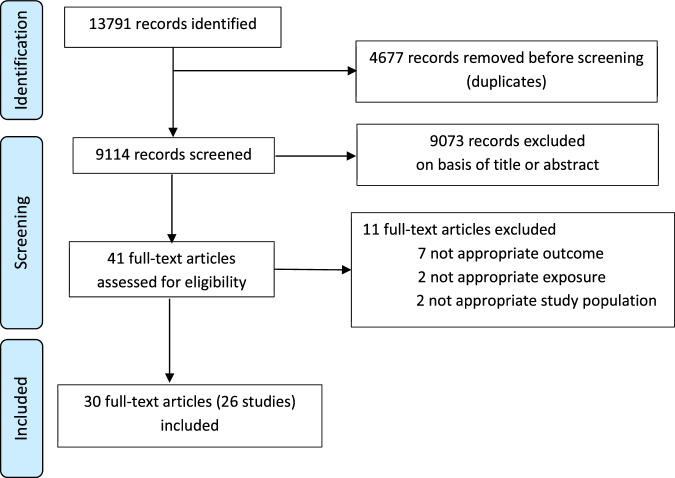
Selection of studies. Flow diagram of identified, screened and included studies and articles, according to PRISMA guidelines.

In total, 9114 records were identified. Of these, 30 (accounting for 26 studies) fulfilled the inclusion criteria and were included in the final analysis^21–50^ (Fig. 1). The characteristics of the studies are summarised in Table 1. Fourteen studies (16 publications) were done in infants and 12 (14 publications) in adults. Nine publications included less than 50 participants, 16 between 50 and 200 and 5 more than 200 participants. Most publications were from studies done in Asia (16 publications), mainly India, Bangladesh, and China, followed by Europe (6 publications), Africa (5 publications), North America (5 publications), New Zealand (2 publications), and South America (1 publication). Most of the studied vaccines were oral: rotavirus (ORV, 8 publications), poliovirus (OPV, 4 publications), cholera (OCV, 2 publications) and *Salmonella typhi* (1 publication). The remainder were parenteral: SARS-CoV-2 (7 publications), PCV, (3 publications), IIV (2 publications), and meningococcus C polysaccharide vaccine (MenC), poliovirus (IPV), tetanus, pertussis, DTaP/Hib (all together), hepatitis B (HBV) vaccines (1 publication each). To assess vaccine responses, most studies measured serum or plasma immunoglobulin (Ig) G (18 publications) or IgA (10 publications, all to ORV or OCV), followed by T-cell responses (5 publications), intestinal IgA (2 publications, OPV) and saliva IgG (1 publication). Stool samples were collected at different time points, but all studies included at least one sample before the first vaccine dose. Studies in infants collected the first stool sample between birth and 6 weeks of age (except one study investigating OPV in which stool samples were collected at 6 months of age). The stool analysis techniques used included 16S rRNA gene sequencing (18 publications), bacteria-specific polymerase chain reaction (PCR) (7 publications), shotgun metagenomic sequencing (10 publications), Human Intestinal Tract Chip (2 publications) and bacterial culture (1 publication). Seven publications used more than one technique.

**Table 1:** Summary of findings of studies investigating the influence of the intestinal microbiota on vaccine responses (significant findings are indicated in bold)

*n*	Study design^#^ Level of evidence^##^	Vaccine and schedule Age (adult studies)	Time stool collected	Stool analysis technique	Time vaccine response measured	Main findings	Author Country Publication year
**Children**							
341	Multicentre birth cohort Level 4	ORV (pentavalent) 6, 10 w	6, 10 w	Shotgun metagenomic sequencing, NovaSeq 6000 (*Illumina)*,	6, 14 w	ORV responders (serum IgA at ≥20 IU/ml or >4x increase of titre): • **lower alpha (Shannon index,** ***p*** < **0.05) and beta diversity (Bray–Curtis distance,** ***p*** < **0.005) among seronegative infants** No differences in prevalence or abundance of individual genera	Cunningham‑Oakes^42^ India, Malawi 2023
72	Multicentre (2 primary care clinics) birth cohort Level 4	DTaP/Hib and PCV13 2, 4, 6, 12 m	2 m	Untargeted metagenomic sequencing HiSeq *(Illumina)*	12 m	DTaP/Hib and PCV plasma or serum IgG. Responders if >4x titre values protective thresholds (categorical) or as continuous • **lower alpha diversity (evenness of genera,** ***p*** = **0.04) for continuous IgG (for DTaP/Hib)** No differences in prevalence or abundance of individual genera	Shaffer^43^ USA 2023
472	Multicentre cohort Level 4	IPV or OPV, TT and pertussis Country-specific schedules	1, 2, 3 m	qPCR for *Bifidobacterium infantis* and *B. longum*	15 m	Serum neutralising IgG log_2_ titres for poliovirus, tetanus and pertussis: • no association between absolute abundance and early colonisation of *B. infantis* or *longum* with tetanus and pertussis IgG titres • **negative association between abundance of** ***B. infantis*** **and polio IgG titre (*p*** < **0.05)**	Colston^23^ Bangladesh, Pakistan, Tanzania 2022
120	Single centre cohort Level 4	PCV10: 2, 3, 4, 11 m MenC: 12 m	0, 7, 14 d 1, 2, 4, 6, 9, 12 m	qPCR for *E. coli*, *Klebsiella*, *Enterococcus* plus VetMAX™ MastiType Multi Kit (samples at 7 d) 16S rRNA gene sequencing, V4, MiSeq (*Illumina*), RDP and SILVA v119 Subset (*n* = 20 at 7 d): shotgun metagenomic sequencing, NovaSeq (*Illumina*), MetaPhlAn2	PCV:12 m MenC: 18 m	Saliva MenC-specific IgG levels (*n* = 66). High responses associated with' : • no association with alpha diversity (Shannon index), overall composition (Bray–Curtis index) and observed number of species • **higher relative abundance of Lachnospiraceae at 2** **m and** ***Pseudobutyrivibrio***, ***Lachnospira*** **and** ***Roseburia*** **at 12** **m (adjusted** ***p*** < **0.05)**. • **lower relative abundance of Bifidobacteriaceae,** ***Veillonella*** **and** ***Klebsiella*** **at 2** **m (adjusted** ***p*** < **0.05)**. Saliva pneumococcal 6B serotype-specific IgG (*n* = 101). High response associated with (stools from 2 m): • no association with alpha (Shannon index), or beta diversity (Bray–Curtis index), **inverse correlation with number of species**. • **higher relative abundance of** ***Esch/Shigella***, ***Bifidobacterium***, ***Bacteroides*, several** ***Ruminococcaceae*** **OTUs, and** ***Streptococcus bovis*** **(adjusted** ***p*** < **0.05)** • **lower relative abundance of several Bacillota OTUs,** ***Enterobacteriaceae***, ***Prevotella***, ***Bifidobacterium bifidum*** **and** ***Garnerella*** **(adjusted** ***p*** < **0.05)**.	deKoff^24^ Netherlands 2022
155	Multicentre cohort Level 4	PCV13 and TT 2, 4, 6 m	6 w	16S rRNA gene sequencing, V4, V5, MiSeq (*Illumina*), SILVA Subset (*n* = ?): shotgun metagenomic sequencing, NextSeq (*Ilumina*), MetaPhlAn3	12 m	Above median responses to PCV (serum IgG) • no association with beta diversity (Bray–Curtis dissimilarity) • no association with abundance of specific taxa Above median responses to TT (serum IgG) • no association with beta diversity (Bray–Curtis dissimilarity). • **lower abundance** ***Aeriscardovia aeriphila*** **(adjusted** ***p*** < **0.05)**	Moroishi^33^ USA 2022
158	Single centre cohort, nested in RCT (hygiene and feeding measures) Level 4	ORV (monovalent)6, 10 w	4, 12 w	Shotgun metagenomic sequencing, HiSeq 2500 or HiSeqX (*Illumina*), MetaPhlAn3	4, 12 w (median 43 d)	ORV responders (serum IgA > 20 UI/mL) • no difference in alpha diversity (Shannon index and number observed species), beta diversity (Bray–Curtis distance), and functional composition • **higher relative abundance of** ***Bacteroides thetaiotaomicron*** **and** ***Slackia isoflavoniconvertens*** **(*p*** < **0.05)**	Robertson^39^ Zimbabwe 2021
86	Multicentre birth cohort Level 4	ORV (pentavalent) India, Malawi: 6, 10 w UK: 8, 12 w	6, 10 w 8, 12 w	16S rRNA gene sequencing, V3-V4, HiSeq or MiSeq (*Illumina*), SILVA v132	6, 14 w 8, 16 w	ORV responders (serum IgA at ≥20 IU/ml or >4x increase of titre): • **UK:** no difference in alpha diversity (Shannon index) or beta diversity (Bray–Curtis distance) • **India & Malawi: lower alpha (Shannon index,** ***p*** < **0.05) and beta diversity (Bray–Curtis distance,** ***p*** < **0.005) among seronegative infants** No differences in prevalence or abundance of individual genera	Parker^36^ UK, India, Malawi 2021 (same as^42^)
45	Single centre cohort Level 4	ORV (pentavalent) 2 m	2 m	16S rRNA gene sequencing, V4, MiSeq (*Illumina*), Greengenes	3 m	ORV responders (serum IgA 4-fold increase): • no association with alpha diversity (Shannon index) or richness • **higher relative abundance of** ***Eggerthella*** **(*p*** = **0.02, FDR** = **1)** • **lower relative abundance of** ***Enterobacteriaceae*** **(*p*** = **0.03, FDR** = **1)**	Fix^26^ Nicaragua 2020
107	Single centre cohort nested in RCT (different OPV vaccination schedules) Level 4	OPV 2, 3, 4 m IPV-bOPV-bOPV or IPV-IPV-bOPV or IPV-IPV-tOPV	3, 3.5, 4 m	16S rRNA gene sequencing, V4, MiSeq (*Illumina*), SILVA	3, 3.5, 4 m	Responders (faecal IgA≥20 IU/ml or 4x increase of titre) at 0 d: • **lower alpha (Shannon index,** ***p*** = **0.0478) and beta diversity (UniFrac distance,** ***p*** = **0.024)** • **lower relative abundance of Bacillota (*p*** < **0.001) and higher relative abundance of Actynomycetota (*p*** < **0.01)** • **lower relative abundance of** ***Clostridia*** **(*p*** < **0.001) and higher relative abundance of unidentified Actynomycetota (*p*** < **0.05)** • **lower relative abundance of** ***Clostridiales*** **(*p*** < **0.001) and higher relative abundance of unidentified** ***Bifidobacteriales*** **(*p*** < **0.05)** • **lower relative abundance of** ***Clostridium*** **sensu stricto (*p*** < **0.01)**	Zhao^41^ China 2020
291	Single centre cohort nested in RCT (vitamin A supplement) Level 4	OPV: 0, 6, 10, 14 w TT-HBV: 6, 10, 14 w BCG: 0 w	6, 11, 15 w	*Bifidobacterium*-specific PCR and T-RFLP 16S rRNA gene sequencing, V4, MiSeq (*Ilumina*), Greengenes May 2013	6, 15 w, 2 y	Higher BCG-specific T-cell responses at 15 w and 2 y: • **higher relative abundance of** ***Bifidobacterium*** **at 6,11,15 w (*p*** < **0.05)** • **higher relative abundance of** ***B. longum*** **and** ***B. longum*** **subspecies** ***infantum*** **at 6w (*p*** < **0.05) (only associated with response at 15 w)** Higher tetanus-specific T cell responses at 15 w and 2 y: • **higher relative abundance of** ***Bifidobacterium*** **at 6,11,15 w (*p*** < **0.05)** • **higher relative abundance of** ***B. longum*** **and** ***B. longum*** **subspecies** ***infantum*** **at 6w (*p*** < **0.05) (only associated with response at 15 w)** Higher tetanus-specific IgG levels in serum at 15 w and 2 y: • **higher relative abundance of** ***Bifidobacterium*** **at 6,11,15 w** (***p*** < **0.05) (only associated with response at 2** **y)** • **higher relative abundance of** ***B. longum*** **at 6w** (***p*** < **0.05) (only associated with response at 15 w)** Higher HepB-specific T cell responses at 15 w: • **higher relative abundance of** ***Bifidobacterium*** **at 6,11,15 w (*p*** < **0.05)** • **higher relative abundance of** ***B. longum*** **at 6w (*p*** < **0.05)** Higher HepB-specific IgG levels in serum at 15 w or 2 y: • **lower relative abundance of** ***B. breve*** **at 6w (*p*** < **0.05) (only associated with response at 15 w)** • **lower relative abundance of** ***B. longum*** **subspecies** ***longum*** **at 6w (*p*** < **0.05) (only associated with response at 2** **y)** Higher polio-specific faecal IgA at 2 years (serotypes 1–3) • **higher relative abundance of** ***Bifidobacterium*** **at 6,11,15 w (*p*** < **0.05)** Higher polio-specific plasma IgA at 2 years • **higher relative abundance of** ***Bifidobacterium*** **and** ***B.longum*** **at 6w (*p*** < **0.05) (Serotype 3)** • **lower relative abundance of** ***B.breve*** **at 6w (*p*** < **0.05) (Serotype 1)** Higher polio-specific plasma IgG at 15 w or 2 y • **higher relative abundance of** ***Bifidobacterium*** **at 6,11,15 w and** ***B.bifidum*** **at 6 w (*p*** < **0.05) (all strains, only associated with response at 2 years)**. • **lower relative abundance of** ***B.longum longum*** **at 6w (*p*** < **0.05) (all strains, only associated with response at 2 years)**.	Huda^31^ Bangladesh 2019
120	Single centre cohort nested in RCT (azithromycin on immunogenicity of OPV) Level 4	OPV (mOPV3) Single dose at 6 to 11 m	2w before and on vaccination day	16S rRNA gene sequencing, V4, MiSeq (*Ilumina*), RDP Pathogens: real-time PCR Taqman array card	3w after vaccination day	Responders (serum neutralising IgG ≥1:8): • no difference in relative abundance of specific bacterial taxa **Non-vaccine-shedders had higher numbers of bacterial taxa (number of OTUs,** ***p*** < **0.05),** **higher diversiy (Shannon index,** ***p*** < **0.05) and higher relative abundance of** ***Clostridia*** **(*p*** = **0.04)**	Praharaj^38^ India 2019
30	Single centre matched case-control, nested in RCT (different ORV dosing) Level 3	ORV (movalent) 6, 10, 14 w or 6, 10 w or 10, 14 w	Before vaccination and 7 d after last dose	Human Intestinal Tract Chip (Microarray)	Before vaccination and 28 d after last dose	Responders (serum IgA ≥ 20 IU/mL): • **higher relative abundance of Bacillota (*Clostridium*** **cluster XI) (*p*** = **0.02, FDR** **=** **0.36) and Pseudomonadota (*p*** **=** **0.04, FDR** = **0.36)** • **higher relative abundance of Gram negative bacteria related to** ***Serratia*** **(*p*** **=** **0.01, FDR** = **0.19) and** ***E. coli*** **(*p*** < **0.01, FDR** **=** **0.05)**	Harris^27^ Pakistan, Netherlands 2018
325	Single centre case-control, nested in RCT (zinc and probiotic supplement) Level 3	ORV (movalent) plus OPV 6, 10 w	6, 10 w	Pathogens: real-time PCR Taqman array card 16S rRNA gene sequencing, V4, MiSeq (*Ilumina*), RDP	6, 14 w	Responders (serum rotavirus IgA 20 ≥ IU/ml or >4x increase of titre): • no differences in composition or diversity (Shanon index for alpha diversity, Unifrac distances for beta diversity) • **more likely to harbour** ≥ **1 bacterial enteropathogen (26 vs 13%,** ***p*** = **0.006) at dose 1 (not dose 2)** **Rotavirus shedding was associated with a higher number of bacterial taxa** (greater OTU count)	Parker^37^ India 2018
78	Single centre matched case-control nested in RCT (different ORV dosing) Level 3	ORV (monovalent) 6, 10, 14 w or 6, 10 w	6 w	Human Intestinal Tract Chip (Microarray)	12 w	Responders (serum IgA ≥ 20 IU/mL): • no difference in alpha diversity (Shannon index) (*p* = 0.87) • **higher Enterobacteria-Bacteroides ratio (*p*** = **0.04)** • **lower relative abundance of Bacteroidota (FDR** **=** **0.003), and several Bacteroides and Prevotella species (FDR** **=** **0.03-0.1)** • **higher relative abundance of Bacillota (FDR** **=** **0.027) and** ***Streptococcus bovis*** **(FDR** **=** **0.008)**	Harris^28^ Ghana 2016
48	Single centre cohort nested in RCT (vitamin A supplement) Level 4	OPV: 0, 6, 10, 14 w TT-HBV: 6, 10, 14 w BCG: 0 w	6, 11, 15 w	*Bifidobacterium*-specific PCR and T-RFLP 16S rRNA gene sequencing, V4, MiSeq (*Ilumina*), ns	15 w	Higher BCG-, polio-, TT- and HBV-specific T cell responses: • **higher relative abundance of Actynomycetota (*B. longum)*** **(except HBV), Bifidobacteriales and Bifidobacteriaceae (except HBV, OPV), and** ***Bifidobacterium*** **and** ***Corynebacterium*** **(only TT), and Coriobacteriales and Coriobacteriaceae (only OPV) (*p*** < **0.05)**. • **higher relative abundance of Porphyromonadaceae (OPV), Enterococcaceae (OPV and BCG) and Enterococcus (BCG) (*p*** < **0.05)**. • **higher relative abundance of β-Proteobacteria and Burkholderiales (BCG); and Pseudomonaceae (TT) (*p*** < **0.05)** • **lower relative abundance Bacillota, Clostridia, Clostridiales and Lactobacillaceae (TT); Veillonella (OPV) and Lactococcus (OPV and BCG) (*p*** < **0.05)**. • **lower relative abundance of Pseudomonadales, Moraxellaceae (except OPV) and Acinetobacter (except HBV and OPV); γProteobacteria, Enterobacteriales, Enterobacteriaceae,** ***Escherichia*** **and** ***Shigella*** **(TT), Pseudomonaceae (OPV) (*p*** < **0.05)** Higher HBV-, polio-, and TT-specific serum IgG levels and DTH skin-test response for tuberculosis: • **higher relative abundance of Actinomycetales (BCG and TT) and** ***Actinomyces*** **(TT); Micrococcaceae and** ***Rothia*** **(BCG and HBV); Bidifobacteriales, Bifidobacteriaceae (BCG) and** ***Bifidobacterium*** **(BCG and OPV) (*p*** < **0.05)**. • **lower relative abundance of Prevotellaceae and** ***Prevotella*** **(BCG) (*p*** < **0.05)**. • **lower relative abundance of Staphylococcaceae (OPV) and** ***Staphylococcus*** **(TT); Carnobacteriaceae and Lachnospiraceae (HBV); Clostridium XI, Finegoldia, Peptoniphilus, Megasphaera (BCG) (*p*** < **0.05)**. • **lower relative abundance of Pseudomonadota, βProteobacteria and γProteobacteria, Enterobacteriales and Enterobacteriaceae (BCG); Pseudomonales, Moraxellaceae and** ***Acinetobacter*** **(OPV)**.	Huda^32^ Bangladesh 2014 (subgroup of^31^)
20	Single centre cohort nested in RCT (fermented vs standard infant formula) Level 3	DTaP-IPV/Hib: 8, 12, 16 w 9 fed bifidogenic formula	4, 8, 12, 16 w	Bacterial culture Multiplex PCR for *Bifidobacterium*	16 w	Higher polio-specific faecal IgA levels: • **detectable levels of** ***B. longum*** **subspecies** ***B. infantis*** **at 16 w (*p*** < **0.002)**	Mullie^34^ France 2004
**Adults**							
36	Multicentre cohort Level 4	Intramuscular SARS-CoV-2 vaccine (inactivated) 0*, 28 d, 7 m Mean 53 years (IQR: 48-56)	Before 0* d	Shotgun metagenomic sequencing, NovaSeq 6000 (*Illumina)*, MetaPhlAn3	12* m	Serum neutralising antibodies high responders (virus microneutralization assay titre ≥1:10). • **lower relative abundance of** ***Eubacterium rectale*** **(*p*** = **0.002),** ***Collinsella aerofaciens*** **(*p*** = **0.038),** **and** ***Streptococcus salivarius*** **(*p*** = **0.021)**.	Zhang^47^ Hong Kong 2024
14	Single centre cohort Level 4	Intramuscular SARS-CoV-2 vaccine (mRNA BNT162b2 and mRNA-1273) 0*, 21-28 d Mean 30 years (range 18-48)	Before 0* d	16S rRNA gene sequencing, MiSeq (*Illumina*), SILVA	0*, 14, 28-35 d	Higher anti-SARS-CoV-2 spike IgG levels (28-35 d, as continuous) • **higher alpha (Shannon index,** ***p*** = **0.048) and beta diversity (Jaccard distances,** ***p*** = **0.019)** • **higher relative abundance of Desulfobacterota,** ***Bilophila*** **and** ***Oscillospiraceae*** **and** ***Alistipes*** **(FDR** < **0.05)** • l**ower relative abundance of** ***Colidextribacter, Clostridium innocuum, Lachnoclostridium, genus UCG 004 (Lachnospiraceae)*** **and Bacteroides (FDR** **<** **0.05)**	Daddi^48^ USA 2023
75	Single centre cohort nested in RCT for SARS-CoV-2 BNT162b2 vaccine Level 4	Intramuscular SARS-CoV-2 vaccine (mRNA BNT162b2) 0*, 21 d Median 54 years, (IQR: 38-64)	0* d	16S rRNA gene sequencing, MiSeq (*Illumina*), SILVA v132	0*, 35 d	High anti-SARS-CoV-2 spike IgG responders day 35 • **lower alpha (Shannon index, FDRadjusted** = **0.009) and beta diversity (NMDS,** ***p*** < **0.01)** • **higher relative abundance of Bacteroidota (*p*** < **0.01),** ***Bacteroides, Sutterella****,* **and** ***Lachnospiraceae*** **FCS020 group (all** ***p*** < **0.05)** • **lower relative abundance of Bacillota (*p*** < **0.01), several** ***Ruminococcaceae Alloprevotella, Anaerofilum, Succinivibrio, Moryella****,* **and** ***Negativibacillus*** **(all** ***p*** < **0.05)** High spike CD4 + T-cell response • **lower alpha (Shannon index, FDRadjusted** = **0.003) and beta diversity (NMDS,** ***p*** < **0.01)** • **higher relative abundance of** ***Lactobacillaceae*** **(*p*** = **0.01) and** ***Lactobacillus*** **(*p*** = **0.014)** • **lower relative abundance of** ***Akkermansiaceae, Akkermansia, Ruminiclostridium, Hydrogenoanaerobacterium*** **and** ***Marvinbryantia*** **(all** ***p*** < **0.05)**	Ray^50^ Sweden 2023
127	Multicentre cohort Level 4	Intramuscular SARS-CoV-2 vaccine (mRNA BNT162b2 or inactivated) Schedule ns, 2 doses mRNA BNT162b2: median 42 years (IQR: 29-54) Inactivated: median 55 years (IQR: 40-57)	0* to 3d, 28 d after last dose	Shotgun metagenomic sequencing, NovaSeq 6000 (*Illumina)*, MetaPhlAn4	0* to 3d, 28 d, 6 m after last dose	Serological tests: plasma surrogate virus neutralisation test and spike receptor-binding domain IgG at 6 m after 2nd dose High responders to mRNA BNT162b2 • **higher relative abundance of several** ***Lachnospiraceae*** **and other Clostridia,** ***Bifidobacterium adolescentis***, ***B. bifidum***, ***Parasutterella excrementihominis*** **and** ***Fusobacterium ulcerans*** **(*p*** < **0.05)** • **lower relative abundance of Clostridiaceae,** ***Roseburia***, ***Latilactobacillus sakei*** **and** ***Alistipes dispar*** **(*p*** < **0.05)** High responders to inactivated vaccine • **higher relative abundance of several** ***Lachnospiraceae***, ***Oscillospiraceae*** **and** ***Clostridiaceae***, ***Solobacterium,Phocaeicola dorei***, ***Actynomyces*, and** ***Rikenellaceae bacterium*** **(*p*** < **0.05)** • **lower relative abundance of several Bacteroidales,** ***Lachnospiraceae***, ***Oscillospiraceae*** **and** ***Clostridiaceae***, ***Intestinimonas butyticiproducens***, ***Emergencia timonensis*** **and** ***Citrobacter freundii*** **(*p*** < **0.05)**	Peng^49^ Hong Kong 2023
86	Single centre cohort Level 4	Intramuscular SARS-CoV-2 vaccine (mRNA BNT162b2) 0*, 22 d Mean 52 years (range: 20-81)	0* d	16S rRNA gene sequencing, V3, V4, MiSeq (*Illumina*), SILVA v119	24*, 30, 63 d	High anti-SARS-CoV-2 spike IgG responders (value for definition not specified) • No association with alpha diversity (Shannon index) • No association with specific bacterial taxa when adjusted for age, sex and timing of stool sampling High T-cell responders (value for definition not specified) • No association with alpha diversity (Shannon index) • No association with specific bacterial taxa when adjusted for age, sex and timing of stool sampling	Hirota^30^ Japan 2023
138	Multicentre cohort Level 4	Intramuscular SARS-CoV-2 vaccine (mRNA BNT162b2 or inactivated) Schedule ns, 2 doses Median 47 years (range: 18-67)	0* to 3d, 28 d after last dose	Shotgun metagenomic sequencing, NovaSeq 6000 (*Illumina)*, MetaPhlAn3	0* to 3d, 28 d after last dose	Serological tests: plasma surrogate virus neutralisation test and spike receptor-binding domain IgG at 28 d after 2^nd^ dose High responders to mRNA BNT162b2 • **higher relative abundance of** ***Eubacterium rectale***, ***Roseburia faecis***, ***Bacteroides thetaiotaomicron*** **and** ***Bacteroides OM05-12*** **(*p*** < **0.05)** High responders to inactivated vaccine • **higher relative abundance of** ***Bifidobacterium adolescentis*** **(*p*** < **0.05)** • **lower relative abundance of** ***Bacteroides vulgatus***, ***Bacteroides thetaiotaomicro*n and** ***Ruminococcus gnavus*** **(*p*** < **0.05)**	Ng^35^ Hong Kong 2022 (same as^49^)
207	Single centre cohort Level 4	Intramuscular SARS-CoV-2 vaccine (inactivated BBIBP-CorV) 0*, 28 d Median 34 years (IQR: 29-46)	0*, 14, 42 d	Shotgun metagenomic sequencing, NovaSeq 6000 (*Illumina)*	0*, 14, 42 d	High ACE2-RBD inhibiting antibody at day 42 • No association with alpha diversity (Shannon index) • **higher relative abundance of** ***Collinsella aerofaciens, Fusicatenibacter saccharivorans, Eubacterium ramulus*, and** ***Veillonella dispar*** **(*p*** < **0.05)** • **lower relative abundance of** ***Lawsonibacter asaccharolyticus*** **(*p*** < **0.05)**	Tang^45^ China 2022
27	Single centre cohort nested in RCT (new OCV vaccine Phase I) Level 4	OCV (Oral MucoRice CTB) 1 dose Mean 29 years (range 20-40)	0*, 2, 4, 6, 8, 16 w	Shotgun metagenomic sequencing, HiSeq 2500 (*Illumina*)	0*, 2, 4, 6, 8, 16 w	Responders (≥4-fold increase of titre of serum CTB IgA and IgG) • **higher beta diversity (Unifrac distances,** ***p*** = **0.044)**, no difference in alpha diversity • **higher relative abundance of** ***Shigella dysentariae*** **(p** = **0.018),** ***S. flexneri*** **(*p*** = **0.032),** ***S. sonnei*** **(*p*** = **0.037),** ***Anaerobaculum mobile*** **(*p*** = **0.001) and** ***Bacillus licheniformis (p*** = **0.002)** • **lower relative abundance of Bacteroides**	Yuki^46^ Japan 2021
69	Single centre cohort nested in RCT (OCV stored at different temperatures) Level 4	OCV 0* d (sd) or 0, 14 d (td-14) or 0, 30 d (td-30) Mean 29 years (range 18-44)	0* d	16S rRNA gene sequencing, V4, MiSeq (*Illumina*), SILVA v138	All: 0*, 3, 7, 90 d Additionally: 30d (sd); 17, 42, 180 d (td-14); 60, 180 d (td-30)	*V. cholerae* O polysaccharide-specific response (plasma IgA and IgG ≥4-fold change): • no association with gut microbiota diversity (phylum level abundance or alpha diversity by inverse Simpson, and beta diversity by Bray-Curtis distance) • **higher relative abundance of** ***Clostridiales*** **(*Sarcina*** **and** ***Clostridiales*** **sensu stricto species) (*p*** < **0.001)** • **lower relative abundance of** ***Enterobacterales*** **(*p*** < **0.01)**	Chac^22^ Bangladesh 2021
122	Single centre cohort Level 4	IM inactivated trivalent influenza vaccine 1 dose Mean 35 years (range 18-64)	0*, 28 d	16S rRNA gene sequencing, V1-V3, MiSeq (*Illumina*), RDP	0*, 28, 180 d	High responder (≥4-fold increase in serum IgG titre against H1 and H3): • no differences in diversity (Shanon index for alpha diversity, Bray-Curtis index for beta diversity) • **higher relative abundance of Bacillota (several OTUs),** **Alphaproteobacteria,** ***Sutterella***, ***Parabacteroides*** **(H1N1) (*p*** < **0.05)** • **higher relative abundance of Bacillota (several OTUs),** ***Bifidobacterium bifidum***, ***Prevotella*** **and cyanobacteria (H3N2) (*p*** < **0.05)** • **lower relative abundance of Bacillota (several OTUs) and** ***Butyricimonas*** **(H1N1) (*p*** < **0.05)** • **lower relative abundance of Bacillota (several OTUs) and** ***H. parainfluenzae*** **(H3N2) (*p*** < **0.05)** In naive influenza vaccination: high response to H1N1 and H3N2 associated with higher relative abundance of Bacillota (several OTUs) (*p* < 0.05)	Cait^21^ New Zealand 2021
15	Single centre cohort Level 4	HBV 0*, 28, 180 d ns (range: 44-73 years)	-14, 0*, 14 d	16S rRNA gene sequencing, V4, MiSeq (*Illumina*),	-14*, 28, 180, 208 d	Higher Anti-HBsAg plasma IgG at 208 d • **higher relative abundance of** ***Butyricicoccus***, ***Clostridium***, ***Phascolarctobacterium*** **and Lachnospiraceae** **(*p*** < **0.05)** • **lower relative abundance of Bacteroidota,** ***Desulfovibrionales***, ***Bacteroides***, ***Atopobium***, ***Gardenerella***, ***Clostridiales***, ***Dialister***, ***Blautia***, ***Desulfovibrionales*** **and** ***Fusobacterium*** **(*p*** < **0.05)**	Shannon^44^ Canada 2020
125	Single centre cohort (feasibility study) Level 3	IM inactivated trivalent influenza vaccine 1 dose Mean 35 years (range: 18-64)	0*, 28 d	16S rRNA gene sequencing, V4, MiSeq (*Illumina*), RDP	0*, 28, 180 d	No association between intestinal microbiota enterotypes and serum influenza-specific IgG response (not powered for this outcome, feasibility study)	Shortt^40^ N. Zealand 2018 (Same as^21^)
66	Single centre cohort nested in RCT (broad vs narrow spectrum antibiotics vs control) Level 4	ORV (monovalent) 1 dose Mean 24 years (SD: 4-4.5)	0* d and before antibiotic	16S rRNA gene sequencing, V4, MiSeq (*Illumina*), RDP	7*, 14, 28 d	Rotavirus IgA boosting ( ≥ 2-fold increase in rotavirus-specific serum IgA titre) and shedders • **higher relative abundance of Prevotellaceae and Porphyromonadaceae and Ruminococcaceae (*p*** < **0.05)** • **lower relative abundance of Fusobacteriaceae and Erysipelotrichaceae (*p*** < **0.05)**	Harris^29^ Netherlands 2018
13	Single centre non-randomised trial Level 3	Oral *Salmonella* typhi live-attenuated vaccine (Ty21a) Schedule ns *n* = 6: 4 doses *n* = 7: 1 dose Median 26 years (range: 19-50)	- 7, 0*, 2, 4, 7, 10, 14, 28, 42, 56 d	16S rRNA gene sequencing, V1, V2, Roche 454 (*Roche diagnostics*), Greengenes March 2011	- 7, 0*, 2, 4, 7, 10, 14, 28, 42, 56 d	Multiphasic cell-mediated immune response • **more diverse, complex bacterial communities: higher alpha diversity (inverted Simpson index (*p*** < **0.0001), Shannon index (*p*** < **0.0001)) and phylogenetic diversity (*p*** < **0.0001) (mostly within order Clostridiales, predominantly within** ***Lachnospiraceae*** **and** ***Ruminococcaceae*** **(*q*** **value** **<** **0.05).** Responders (≥4x increase of titre of serum IgA and IgG) • no difference in overall community diversity (Shannon, inverted Simpson and phylogenetic diversity)	Eloe-Fadrosh^25^ USA 2013

*BCG* Bacille-Calmette-Guérin vaccine, *CTB* cholera toxin B, *d* days, *DTaP-IPV/Hib* diphtheria-tetanus-acellular pertussis-inactivated poliomyelitis-H. influenzae type b vaccine, *FDR* false discovery rate*, HB*V Hepatitis B vaccine, *IM* intramuscular, *Ig* immunoglobulin (G/A), *IPV* intramuscular poliovirus vaccine, *IQR* interquartile range, *m* months, *MenC* meningococcus C polysaccharide vaccine, *ns* not specified, *OCV* oral cholera vaccine, *OPV* (t/b) oral poliovirus vaccine (trivalent/bivalent), *ORV* oral rotavirus vaccine, *OTU* operational taxonomic unit, *(q)PCR* (quantitavie) polymerase chain reaction, *PCV* pneumococcal conjugate vaccine, *RCT* randomised controlled trial, *RDP* ribosomal database project, *RNA* ribonucleic acid, *SARS-CoV-2* Severe acute respiratory syndrome coronavirus 2, *sd* single dose, *SD* standard deviation, *td(-14/30)* two doses, second after 14 or 30 days, *T-RFLP* terminal restriction fragment length polymorphism, *TT* tetanus vaccine, *UK* United Kingdom, *w* weeks. # They are all prospective studies; * 0 = day 1st dose; ## Oxford Centre for Evidence-Based Medicine 2011 Levels of Medicine **(A/Victoria/4897/2022 (H1N1)pdm09-like strain, A/Darwin/9/2021 (H3N2)-like strain, B/Austria/1359417/2021-like strain, B/Phuket/3073/2013 like strain).

All included publications had an overall risk of bias score (JBI standardised critical appraisal checklist, yes%) over 60% (acceptable quality), and 80% (24/30) of publications had an overall score ≥80% (good quality) (Supplementary Table 2). The most frequent risk of bias was a lack of identification of confounding factors and strategies to address these (present in 57% (17/30) studies). Some studies did not describe clearly how many children completed follow-up or the reasons for lost to follow-up. In some of the adult studies, ≥4-fold increase in antibody levels were used to overcome the difficulty of including seronegative or vaccinated naive subjects.

In total, 87% (26/30) of the publications reported an association between the composition of the intestinal microbiota and vaccine responses, though 3 of them found only an association with alpha or beta diversity and not with relative abundance of specific bacteria. We did not observe important differences between the studies assessing oral vaccines and those assessing parenteral vaccine responses. The findings are summarised in Tables 1 and 2, and in Supplementary Table 3.

**Table 2:**
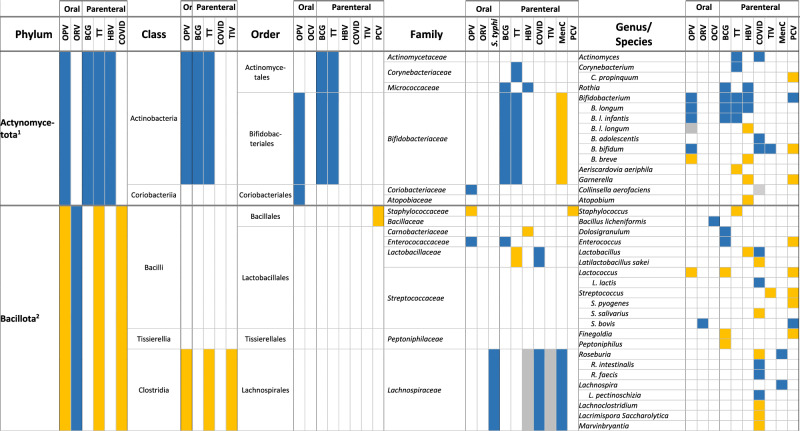

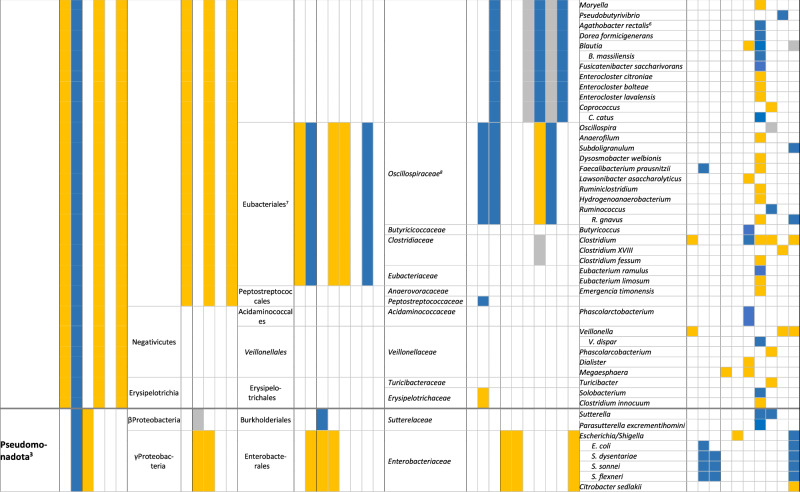

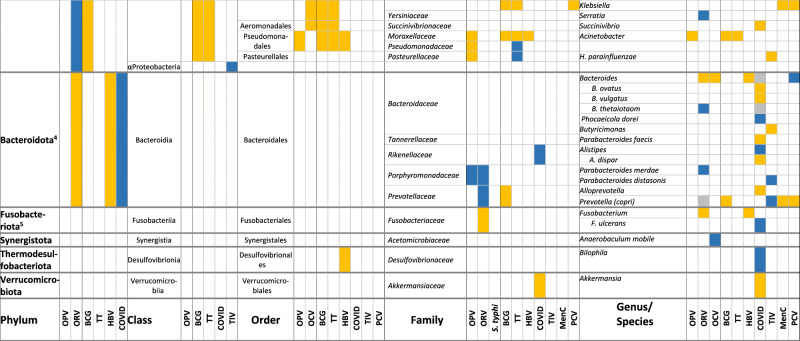
Associations between the composition of the intestinal microbiota at different taxonomic levels and vaccine responses (colour coding: higher relative abundance associated with higher vaccine response (blue); lower vaccine response (yellow), inconsistent findings (grey); all *p* < 0.05)

*BCG* Bacille-Calmette-Guérin vaccine, *COVID* SARS-CoV-2 vaccine, *HBV* HepB vaccine, *MenC* meningococcus C polysaccharide vaccine, *OCV* oral cholera vaccine, *OPV* oral poliovirus vaccine, *ORV* oral rotavirus vaccine, *PCV* pneumococcal conjugate vaccine, *S. typhi*: oral *Salmonella typhi* vaccine, *TIV* trivalent influenza vaccine, *TT* tetanus vaccine

1: Previous Actinobacteria; 2: Previous Firmicutes; 3: Previous Proteobacteria; 4: Previous Bacteroidetes; 5: Previous Fusobacteria; 6: Previous *Eubacterium rectale*; 7: Previous/other: Clostridiales; 8: Previous *Ruminocaccaceae*.

### Oral vaccines

Six publications in infants^26–28,36,37,39,42^ and one in adults^29^ reported on the association between the composition of the intestinal microbiota and serum IgA response to ORV, defining high responders as an IgA titre of ≥20 IU/mL or a ≥4-fold increase in titre (Tables 1 and 2). Five studies used the monovalent ORV vaccine^27–29,37,39^ and three the pentavalent^26,36,42^. Two publications reporting findings from the same study found a lower alpha and beta diversity of the intestinal microbiota among responders, but only in India and Malawi and not in the UK^36,42^. In the other studies, a higher relative abundance of Bacillota, *Ruminococcaceae* and *Peptostreptococcaceae*, *Streptococcus bovis* and *Faecalibacterium prausnitzii* and a lower relative abundance of *Erysipelotrichaceae* and *Clostridium XVII* were associated with ORV vaccine responders (all *p* < 0.05)^27–29^. Furthermore, a higher relative abundance of Pseudomonadota (*Escherichia coli* and *Serratia*), and Bacteroidota (*Porphyromonadaceae* and *Prevotellaceae*, and *Bacteroides thetaiotaomicron*) were associated with responders (*p* < 0.05), while for *Bacteroides* and *Prevotella* contradicting findings were reported^27–29,39^. A lower relative abundance of *Fusobacteriaceae* was also associated with higher vaccine responses (*p* < 0.05)^29^. One study found no association between the diversity or composition of the intestinal microbiota, but responders more often had more than one bacterial enteropathogen (26 vs 13%, *p* = 0.006)^37^.

Responses to OPV were assessed in 5 infant studies^23,31,32,38,41^, one of them using a subgroup of participants from a larger study^31,32^. Vaccine response assessment included polio-specific intestinal IgA, serum and plasma IgA and IgG titres, as well as T-cell responses^23,31,32,38,41^. Only one study found an association between a lower alpha and beta diversity and higher vaccine responses (intestinal polio-specific IgA, *p* < 0.05)^41^. Other studies reported that a higher relative abundance of Actynomycetota (from phylum to family level) and a lower relative abundance of Bacillota (from phylum to species level) and Pseudomonadota (from order to species level) were associated with higher OPV vaccine responses (*p* < 0.05)^31,32,41^. Exceptions to this were an association between a higher relative abundance of *Enterococcaceae* (Bacillota)^32,41^ and lower relative abundance of *Bifidobacterium breve* (Actynomycetota)^31^ with higher OPV vaccine responses (*p* < 0.05). A higher relative abundance of *Prophyromonadaceae* (Bacteroides) was also associated with higher OPV vaccine response (*p* < 0.05)^32^. One study found no association between the relative abundance of specific bacterial taxa in the intestinal microbiota and polio-specific serum IgG responses^38^.

Two studies investigated the association between the composition of the intestinal microbiota and OCV responses (plasma IgA and IgG ≥4-fold change in titre) in adults^22,46^. One study reported a higher beta diversity (UniFrac distances, *p* = 0.044) and vaccine response among high OCV responders^46^. These studies found an association between a higher relative abundance of *Clostridiales*^22^, *Shigella*, *Anaerobaculum mobile* and *Bacillus licheniformis*^46^, a lower relative abundance of *Enterobacteriales*^22^ and *Bacteroides*^46^, and higher memory B cell responses (*p* < 0.05).

Another study in adults found a higher alpha diversity and phylogenetic diversity with a higher relative abundance of *Clostridiales* (*Lachnospiraceae* and *Ruminococcaceae*) was associated with multiphasic cell-mediated immune response after an oral *Salmonella typhi* vaccine (*p* < 0.05). No association between the composition of the intestinal microbiota and serum IgA or IgG response was found^25^.

### Parenteral vaccines

Three infant studies analysed the effect of the composition of the intestinal microbiota on the response to PCV^24,33,43^. The first study found no association between PCV vaccine response and intestinal microbiota diversity or composition^43^. The second study found that a higher relative abundance of *Escherichia/Shigella, Bifidobacterium, Bacteroides*, *Ruminococcaceae*, and *Streptococcus bovis* (adjusted *p* < 0.05) and a lower relative abundance of Bacillota, *Enterobacteriaceae, Prevotella, Bifidobacterium bifidum* and *Garnerella* was associated with higher pneumococcal-specific saliva IgG levels (adjusted *p* < 0.05)^24^. The third study found no association between the beta diversity of intestinal microbiota or the abundance of specific taxa and serum pneumococcal IgG titre^33^. The second study also investigated the association of the intestinal microbiota composition at 2 and 12 months and meningococcal-C saliva IgG titres in response to meningococcus C polysaccharide vaccine given at 18 months. This study found that a higher relative abundance of *Lachnospiraceae* at 2 months and *Pseudobutyrivibrio, Lachnospira* and *Roseburia* at 12 months and lower relative abundance of *Bifidobacteriaceae, Veillonella* and *Klebsiella* at 2 months were associated with higher responses to meningococcus C polysaccharide vaccine (adjusted *p* < 0.05)^24^.

Humoral and T-cell response to SARS-COV-2 vaccine were investigated in seven adult studies^30,35,45,47–50^. Three studies assessed the humoral response to inactivated SARS-CoV-2 vaccines^35,45,47^, and five studies to mRNA vaccines (BNT162b2, one also for mRNA-1273)^30,35,48–50^. Two of the mRNA vaccine studies also assessed T-cell responses^30,50^. A higher relative abundance of several Actynomycetota (*Actynomyces, Bifidobacterium adolescentis and B. bifidum*)^35,49^ was associated with a higher vaccine response, while findings for *Collinsella aerofaciens* were contradictory^45,47^. The phylum Bacillota was negatively associated with SARS-CoV-2 vaccine responses^50^, with mixed findings for its classes and orders (higher relative abundance of *Clostridia*, and lower relative abundance of *Eubacteriales* among higher responders)^45,49,50^. There were also inconsistent findings for most of the remaining families and genera: a higher abundance of *Lactobacillaceae* was associated with higher vaccine responses, but findings for *Lachnospiraceae, Clostridiaceae*, certain *Lactobacillales* and *Oscillospiraceae* were contradictory^45,48–50^. For the Pseudomonadota phylum, a higher relative abundance of *Sutterella* and *Parasutterella excrementihomini*, and a lower relative abundance of *Succinivibrio* and *Citrobacter* were associated with higher vaccine responses^49,50^. While a higher relative abundance of the phylum Bacteroidota and the family *Rikenellaceae* were associated with higher vaccine response, there were contradictory findings for several genus and species within this phylum, including *Bacteroides*, *Parabacteroides, Alistipes* and other^48–50^. Finally, a higher relative abundance of *Bilophila* (a Thermodesulfobacteriota) and a lower abundance of *Akkermansiaceae* and *Akkermansia* (Verrucomicrobiotas) were associated with higher SARS-CoV-2 vaccine responses^48,50^. One study found no association between the alpha diversity of the intestinal microbiota or relative abundance of specific taxa and plasma-specific IgG responses to the mRNA BNT162b2 vaccine^30^.

One study in adults analysed the association between intestinal microbiota and serum influenza-specific IgG after intramuscular IIV, with results reported in two manuscripts^21,40^. The first report classified intestinal microbiota findings into enterotypes and found no association with these enterotypes and influenza-specific IgG responses^40^, stating that the study was underpowered. The second report did an ad hoc analysis on the effect of fibre intake on humoral response to IIV and found that a higher relative abundance of Alphaproteobacteria, certain Bacillota (*Clostridiales, Ruminococcaceae, Ruminococcus*), and Bacteroides (*Parabacteroides* and *Prevotella*), *B. bifidum and Sutterella*, and a lower relative abundance of several Bacillota OTUs (including *Clostridia*), *H. parainfluenzae* and *Butyricimonas* were associated with higher serum antibody responses to H1N1 or H3N2 (all adjusted *p* < 0.05), with contradicting findings for *Lachnospiraceae* and *Oscillospora*. In this study, no association was found between the alpha or beta diversity of the intestinal microbiota and vaccine responses^21^.

Vaccine response to tetanus toxoid vaccine in relation to intestinal microbiota was assessed in three infant studies (serum IgG and T-cell responses)^23,31–33^. A multicentre prospective birth study including 472 infants found no association between the relative abundance of *Bifidobacterium infantis* and *B. longum* and tetanus serum IgG levels^23^. A birth cohort study found that a lower relative abundance of *Aeriscardovia aeriphila* was associated with higher serum IgG responses to tetanus toxoid vaccine^33^. Results from 291 infants from Bangladesh were published in two publications. Stool samples were analysed at three different time points (6, 11, 15 weeks), and serum tetanus-specific IgG and T-cell responses were measured at 15 weeks and 2 years of age. A higher relative abundance of *Bifidobacterium* and *B. longum* was associated with higher T-cell responses and serum IgG titres at different time-point combinations^31^. In a sub-sample of 48 infants, a higher relative abundance of Actynomycetota (*Bifidobacteriales*, *Bifidobacteriaceae, Corynebacterium, Bifidobacterium)* and *Pseudomonadaceae* and a lower relative abundance of certain Bacillota (Clostridia, *Clostridiales* and *Lactobacillaceae)*, and Gammaproteobacteria *(Pseudomonadales, Enterobacteriales, Moraxellaceae, Enterobacteriaceae, Escherichia, Shigella* and *Acinetobacter)*, were associated with higher T-cell responses to tetanus. A higher relative abundance of *Actinomycetales* and *Actinomyces*, and a lower relative abundance of *Staphylococcus* were also associated with higher serum IgG response to tetanus^32^.

A birth cohort in the USA, studied the humoral response to all vaccine components in the DTaP/Hib vaccine^43^, and reported a negative correlation between alpha diversity (evenness of genera, *p* = 0.04) and median IgG DTaP/Hib titres, but no association with relative abundance of specific intestinal bacterial taxa^43^.

Two studies investigated responses to HBV vaccine, one study in infants^31,32^ and one in adults^44^. Among infants, a higher relative abundance of *Bifidobacterium* and *B. longum* and lower relative abundance of *Pseudomonales* and *Moraxellaceae* were associated with higher T-cell responses and a higher relative abundance of *Micrococcaceae* and *Rothia*, and lower relative abundance of *Carnobacteriaceae, Lachnospiraceae*, *B. breve* and *B. longum* were associated with higher serum IgG titres^31,32^. In adults, a higher relative abundance of *Butyricicoccus* and *Phascolarctobacterium*, and a lower relative abundance of *Clostridiales* and *Bacteroides* (all *p* < 0.05) were associated with higher anti-HBsAg plasma IgG in a multiomics model^44^.

This same study in infants also investigated the response to BCG vaccine^31,32^, and reported that a higher relative abundance of Actynomycetota (*Bifidobacteriales, Bifidobacteriaceae*), certain Bacillota (*Enterococcaceae, Enterococcus*) and Betaproteobacteria (*Burkholderiales*), and a lower abundance of certain Gammaproteobacteria (*Pseudomonales*, *Moraxellaceae* and *Acinetobacter*) were associated with higher T-cell responses. Furthermore, a higher relative abundance of certain Actynomycetota (*Actinomycetales, Micrococcaceae, Rothia, Bidifobacteriales, Bifidobacteriaceae* and *Bifidobacterium*) and a lower abundance of several Bacteroidota (*Prevotellaceae* and *Prevotella*) and Bacillota OTUs were associated with positive delayed-type hypersensitivity skin-test responses (*p* < 0.05)^31,32^.

One cohort study in infants found an association between detectable levels of *B. longum* subspecies *infantis* and higher polio-specific intestinal IgA titres as a response to IPV (*p* < 0.002)^34^. Two of the studies mentioned above which assessed response to OPV, also used IPV as a combination with OPV in different schedules, or according to country schedule (see above)^23,41^.

We assessed differences between the associations between the gut microbiota relative abundance of specific bacterial taxa and vaccine responses among infants (Supplementary Table 4) and adults (Supplementary Table 5). For the Actynomycetota phylum, there was more evidence of an increased relative abundance among higher vaccine responders in infant studies, with some exceptions such as *Gardnerella* and some *Bifidobacterium* species. In adults, *Bifidobacterium* showed also a positive association with vaccine response. Among the Bacillota phylum, findings were variable for both infants and adults, though more infant studies reported a lower relative abundance of Bacillota among higher vaccine responders compared to adults. A lower relative abundance of Pseudomonota (from phylum to family) was associated with vaccine response in infants, while adult studies reported positive associations more frequently. However, *Escherichia* and *Shigella* genus and species were also positively associated with vaccine response among infants. Bacteroidota phylum bacteria findings were very variable, with a general negative association with vaccine response for Bacteroides genus and species among adults.

## Discussion

Understanding the underlying causes for the variability in vaccine effectiveness among individuals is crucial to improving vaccine performance. Our systematic review shows that the composition of the intestinal microbiota can impact responses to oral and parental vaccines, affecting both humoral and cellular responses. Despite some variability in results among studies, in general, there was a beneficial effect of Actynomycetota (particularly *Bifidobacterium*), and a detrimental effect of Pseudomonadota (especially Gammaproteobacteria) on vaccine responses. The alpha and beta diversity of the intestinal microbiota was not strongly associated with differences in vaccine responses.

Three possible immunological mechanisms have been proposed to explain how the intestinal microbiota modulates vaccine responses: provision of natural adjuvants, modulation of antigen-presenting cells by microbial metabolites, and presence of cross-reactive antigens encoded in the microbiota^14^. Certain microbiota components, such as lipopolysaccharide or flagellin, have been shown to act as vaccine adjuvants by activating antigen-presenting cells through pattern-recognition receptors (PRRs)^51,52^. The microbiota has also been shown to enhance different antigen-presenting cells, such as dendritic cells^53,54^, macrophages^52^ or intestinal epithelial cells^55^. Memory CD4 + T cells encoded for specific pathogen antigens have been found in individuals not previously exposed to these pathogens^56^, and CD4 + T cells reactive to intestinal microbiota can also be found in humans^57^. Some researchers have therefore hypothesised that some of these CD4 + T cells may cross-react to certain epitopes shared between the intestinal microbiota and pathogen antigens present in vaccines^14^. The evidence supporting these potential immunological mechanisms is sometimes contradictory and based mainly on mouse models. A potential issue to consider is that variations in the microbiome may represent a marker of poor vaccine response rather than the cause: individuals with a weaker immune response to vaccines may have specific microbiome profiles, reflecting an association rather than a causal relationship.

Nonetheless, animal studies provide strong evidence for the impact of the composition of the intestinal microbiota on vaccine responses. Antibiotic-treated infant mice mount reduced humoral responses to common licensed live and adjuvanted vaccines^51^. Similarly, pups born to mothers treated with antibiotics show impaired humoral responses to the model antigen ovalbumin^58^. In relation to the role of the activation of PRRs, mice deficient in Toll-like receptor 5 (TLR5) or in nucleotide-binding oligomerization domain-containing (Nod) 2 (both PRRs), and germ-free mice show impaired antibody responses to influenza, IPV and cholera toxin vaccines, with a restored response after bacterial reconstitution^52,59^. However, this reduced humoral response in TLR5-deficient mice depends on the type of vaccine and is not observed after adjuvanted and live vaccines^52^. Some of these studies reported a rescued vaccine response after the intestinal microbiota was restored, especially when restored with flagellated *Escherichia coli*^51,52^.

Studies in mice have also reported differences between adult and infant mice, with no impaired vaccine response in antibiotic-treated adult mice^51,52^. In our systematic review, an effect of the intestinal microbiota on vaccine responses was reported in both adults and infants, though none included both age groups in the same study. The only vaccine investigated across both age groups, albeit in separate studies, was the ORV. Variations in the specific bacterial taxa associated with differences in vaccine response were observed between adults and infants, as well as between different studies in infants^26–29,36,37,39^, precluding definitive conclusions. Further studies including different age groups using the same methods and vaccine are needed to establish the impact of age on the intestinal microbiota on vaccine responses in humans.

A key characteristic of intestinal microbiota is that it can be modified through interventions. Studies have shown how the use of probiotics, prebiotics, and antibiotics modify the diversity and composition of intestinal microbiota^60^. A systematic review on the effect of probiotics on vaccine response, reported that half of the 26 included randomised placebo-controlled trials (RCTs), showed a beneficial effect, especially for oral vaccines and parenteral influenza vaccine^61^. Again, study designs, study vaccine, dosing and timing of the probiotic varied between studies, making it difficult to draw firm conclusions^61^. Also, some of the RCTs did not report previous exposure to antibiotics or excluded participants exposed to antibiotics. Studies assessing the effect of probiotics in infants and adults with disrupted intestinal microbiotas would be highly relevant but currently lacking. Further RCTs in children published since the review have continued to report both positive (for *Haemophilus influenzae* type B vaccine)^62^ or no associations (pneumococcus, diphtheria, tetanus and pertussis vaccines)^62,63^ between probiotic administration and vaccine responses. In adults, a meta-analysis on the effect of pre- and probiotics on immune response to influenza vaccines that included 20 RCTs concluded that their use was associated with an improved response to H1N1, H2N3 and B influenza strains^64^. As discussed above, antibiotic-treated mice have a reduced humoral response to influenza vaccine^52^. However, few human clinical studies have assessed the effect of antibiotics on vaccine responses through disruption of the intestinal microbiota. A retrospective study in children, showed an inverse dose-dependent effect of antibiotic exposure on antibody responses to infant vaccines (DTaP, Hib, IPV and PCV), though intestinal microbiota changes were not assessed^65^. Nonetheless, a trial on the effect of azithromycin on OPV vaccine response in Indian infants, did not improve vaccine immunogenicity despite reducing pathogenic intestinal bacteria prevalence, environmental enteropathy and reducing the relative abundance of Pseudomonadota and Verrucomicrobia^66,67^. In adults, a trial showed that antibiotic-driven intestinal microbiota alterations led to a reduced H1N1-specific IgG1 response only among those with low pre-existing titres, with no effect on participants with high baseline titres or on H3N2 influenza response^68^. Finally, vaccines may themselves generate changes in the intestinal microbiota composition, as was shown in one the studies included in this review, which reported an association between SARS-CoV-2 intramuscular vaccine and a decrease in relative abundance of *Bacteroides caccae* and *Clostridiales*^35^. However, other studies showed no effect of human immunodeficiency virus (HIV)-1 (intranodal and intramuscular), oral typhoid, or BCG vaccines on intestinal microbiota composition^25,69–71^. Overall, there is good evidence that vaccine responses can be modulated by changes in the intestinal microbiota.

The strengths of the study include the comprehensive search strategy with no age, region, language, or time restriction to avoid study selection bias.

Our review is inevitably affected by limitations inherent in the reported studies. While all studies were prospective, predominantly cohort studies, many had small sample sizes (only 3 studies included more than 200 participants), potentially resulting in underpowered analyses. Some studies might be subject to bias from multiple significance testing when they included multiple analyses with several vaccines, multiple humoral and cellular responses, and several time points to measure vaccine response. Thirdly, the heterogeneity in study designs (different vaccines and schedules, timing of stool collection and vaccine response outcome measures, as well as stool analysis techniques), make it difficult to find common patterns. Similarly, results of intestinal microbiota composition were reported at different taxonomic levels. Finally, like all reviews, our analysis depends on published data, which could be affected by reporting and publication biases, potentially leading to the omission of studies with negative findings.

Despite the increasing evidence to support a key role of the composition of the intestinal microbiota on modulating vaccine responses, it is not yet sufficient for translation to clinical practice. Larger multicentre studies applying the same methods and studying the same vaccine responses in different locations and age groups are needed to assess the impact of variations in the intestinal microbiota impact on vaccine response, and their relative importance in relation to other potential vaccine response modulators, such as environmental, perinatal, nutritional and intrinsic host factors^3^. In the present study, we reported both similarities and differences between studies in infants and adults, but this was limited by the use of different vaccines and settings. It would be of interest to do studies in different age groups for vaccines that are used all along the life span, such as SARS-CoV-2 or influenza vaccines. Future studies should investigate other components of the intestinal microbiota such as fungi, archaea and viruses, as there is some evidence for an effect of the intestinal virome on vaccine responses^72^. Further, they should apply more detailed stool analysis techniques, such as whole metagenomic next-generation sequencing, to identify key intestinal microbiota bacteria at the species and strain level. This may lead to adequately powered clinical trials on interventions (probiotics or antibiotics) directed at optimising the balance of key intestinal microbiota bacteria associated with modulating vaccine responses.

Findings from this systematic review support the concept that the composition of the intestinal microbiota impacts vaccine responses. Although further confirmation is needed in more robust studies, these findings have exciting implications for potential microbiota-targeted interventions to optimise vaccine responses.

## Methods

Studies were identified, selected, appraised and synthesised following the Preferred Reporting Items for Systematic Reviews and Meta-Analyses (PRISMA) guidelines for systematic reviews^73^.

### Eligibility criteria

Studies investigating the association between the composition of intestinal microbiota composition and any type of vaccine response (humoral and cellular) in humans were included. For intervention studies in which antibiotics, probiotics and supplements were administered, inclusion was contingent upon the presence of a control group receiving no intervention or that the association between the intestinal microbiota and vaccine responses was observed across all groups. Studies that included participants already infected by the targeted pathogen of the vaccine were excluded.

### Information sources and search strategy

In June 2024, MEDLINE (1946 to present) and Embase (1947 to present) were searched without any language restrictions with the following search terms: microbiota AND stool AND vaccine response. The detailed search terms can be found in Supplementary Table 1. Duplicates were removed. References of retrieved articles were hand-searched for additional publications.

### Study selection, data extraction and quality assessment

Titles and abstracts were screened for eligibility, followed by screening of full texts. Relevant data was extracted into detailed tables, including study characteristics (design, location, and year), population (size and age), exposure (type of vaccine and schedule, timing and technique used for stool sample analysis), outcome measures (timing and technique used for vaccine response analysis), and results (Table 1).

### Quality and risk of bias assessment

The level of evidence of each study was classified according to the 2011 Oxford Centre for Evidence-Based Medicine (OCEBM) Levels of Evidence^74^. Risk of bias was assessed using the 2017 Joanna Briggs Institution (JBI) standardised critical appraisal checklist for case-control and cohort studies^75^.

### Synthesis of results

The findings were summarised into tables, grouped by specific intestinal bacteria (from phylum to species) and vaccine investigated.

## Supplementary information

Supplemental Information

## Data Availability

The data used in this systematic review was extracted from published studies, and we do not possess specific datasets to share. All data generated during this study are included in this published article and its supplementary information files.
