# Supplementary material for: Systematic review of the impact of intestinal microbiota on vaccine responses
Source: NPJ Vaccines. 2024 Dec 20;9:254. doi: 10.1038/s41541-024-01000-0 (PMC11662010; doi:10.1038/s41541-024-01000-0)
Supplement: Supplementary file 1 — Supplemental Information [file 41541_2024_1000_MOESM1_ESM.pdf]

SUPPLEMENTARY MATERIAL

Supplementary Table 1: Specific search strings

A. Medline (Ovid)	
1.Microbiome & pathogens	*Gastrointestinal Microbiome/ or exp *Microbiota/ or exp *Actinobacteria/ or *Bacteroidetes/ or *Proteobacteria/ or *Firmicutes/<br>or<br>(microbiome or microbiota or Actinobacteria or Bacteroides or Bifidobacterium or Enterobacteriaceae or Lactobacillus or Proteobacteria).tw,kf.
2.Location	exp *Feces/ or exp *Intestines/ or exp *Lower Gastrointestinal Tract/<br>or<br>(feces or faeces or fecal or faecal or stool or gut or meconium or intestin* or gastrointestin*).tw,kf.
3.Vaccine response	exp *Vaccines/im or *Vaccine Efficacy/ or *Vaccine Potency/ or *Immunogenicity, Vaccine/ or exp *Antibodies/ or exp *Immunity, Cellular/ or *Immunity, Humoral/ or *Immunity, Mucosal/ or exp *Immune system/im or *Seroconversion/<br>or<br>(vaccin* or immuniz* or immunis* or immunogenicity or antibod* or immunoglobulin or humoral-immunity or cellular-immunity or mucosal-immunity).tw,kf.
4.Combine searches	1 and 2 and 3
5.Exclude Animal studies	(exp animals/ or (rat or rats or mouse or mice or rodent* or swine or porcine or murine or sheep or lamb or lambs or pig or pigs or piglet or piglets or rabbit or rabbits or cat or cats or dog or dogs or cattle or bovine or monkey or monkeys or trout or marmoset or marmosets).ti.) not human*.sh.

6. Combine searches	4 not 5
7. Limit searches	limit 6 to (case reports or comment or editorial or guideline or letter or practice guideline or preprint)
8. Combine searches	6 not 7
**B. Embase (Ovid)**	
1. Microbiome & pathogens	exp *intestine flora/ or exp *microflora/ or exp *Actinobacteria/ or exp *Bacteroidetes/ or exp *Proteobacteria/ or exp *Firmicutes/<br>or<br>(microbiome or microbiota or Actinobacteria or bacteroides or Bifidobacterium or Enterobacteriaceae or lactobacillus or Proteobacteria).kf,dq,tw.
2. Location	*Feces/ or exp *Feces analysis/ or *feces microflora/ or exp *Intestine/ or *Lower Gastrointestinal Tract/<br>or<br>(feces or faeces or fecal or faecal or stool or gut or meconium or intestin* or gastrointestin*).kf,dq,tw.
3. Vaccine response	exp *Vaccine/ or *vaccine immunogenicity/ or exp *Antibody/ or exp *Cellular immunity/ or exp *Humoral Immunity/ or *Mucosal Immunity/ or exp *Microbial immunity/<br>or<br>(vaccin* or immuniz* or immunis* or immunogenicity or antibod* or immunoglobulin or humoral-immunity or cellular-immunity or mucosal-immunity).kf,dq,tw.
4.Combine searches	1 and 2 and 3
5. Animal studies	(rat or rats or mouse or mice or rodent* or swine or porcine or murine or sheep or lamb or lambs or pig or pigs or piglet or piglets or rabbit or rabbits or cat or cats or dog or dogs or cattle or bovine or monkey or monkeys or trout or marmoset or marmosets).ti. and animal experiment/<br>or<br>Animal experiment/ not (human experiment/ or human/)

6. Combine searches	4 not 5
7. Limit searches	case report/<br>or<br>limit 6 to (conference abstract or conference paper or "conference review" or editorial or letter or "preprint (unpublished, non-peer reviewed)")
8. Combine searches	6 not 7
**C. Medline (PubMed) - key word search only**	
1. Microbiome & pathogens	“microbiome”[Title/Abstract] OR “microbiota”[Title/Abstract] OR “Actinobacteria”[Title/Abstract] OR “bacteroides”[Title/Abstract] OR “Bifidobacterium”[Title/Abstract] OR “Enterobacteriaceae”[Title/Abstract] OR “lactobacillus”[Title/Abstract] OR “Proteobacteria”[Title/Abstract] OR “intestine-flora” [Title/Abstract] OR “intestinal-flora” [Title/Abstract] OR “microflora” [Title/Abstract]
2. Location	"feces"[Title/Abstract] OR "faeces"[Title/Abstract] OR "fecal"[Title/Abstract] OR "faecal"[Title/Abstract] OR "stool"[Title/Abstract] OR "gut"[Title/Abstract] OR "meconium"[Title/Abstract] OR "intestin*"[Title/Abstract] OR "gastrointestin*"[Title/Abstract]
3. Vaccine response	"vaccin*"[Title/Abstract] OR "immuniz*"[Title/Abstract] OR "immunis*"[Title/Abstract] OR "immunogenicity"[Title/Abstract] OR "antibod*"[Title/Abstract] OR "immunoglobulin"[Title/Abstract] OR "humoral immunity"[Title/Abstract] OR "cellular immunity"[Title/Abstract] OR "mucosal immunity"[Title/Abstract] OR “seroconversion”[Title/Abstract]
4. Only non-Medline journals or e-Pubs (filter in All Fields)	NOTNLM OR publisher[sb] OR inprocess[sb] OR pubmednotmedline[sb] OR indatareview[sb] OR pubstatusaheadofprint
5. Combine searches	#1 AND #2 AND #3 AND #4

6. Exclude animal studies (filter in All fields)	("Animal" OR "animals" OR "rat" OR "rats" OR "mouse" OR "mice" OR "rodent*" OR "swine" OR "porcine" OR "murine" OR "sheep" OR "lamb" OR "lambs" OR "pig" OR "pigs" OR "piglet" OR "piglets" OR "rabbit" OR "rabbits" OR "cat" OR "cats" OR "dog" OR "dogs" OR "cattle" OR "bovine" OR "monkey" OR "monkeys" OR "trout" OR "marmoset" OR "marmosets") NOT ("human" OR "humans" OR "patient" OR "patients" OR "newborn*" OR "baby" OR "babies" OR "neonat*" OR "infan*" OR "toddler*" OR "pre-schooler*" OR "preschooler*" OR "kindergarten" OR "boy" OR "boys" OR "girl" OR "girls" OR "child" OR "children" OR "childhood" OR "adolescen*" OR "pediatric*" OR "paediatric*" OR "youth*" OR "teen" OR "teens" OR "teenage*" OR "school-aged*" OR "school-child*" OR "school-girl*" OR "school-boy*" OR "schoolgirl*" OR "schoolboy*" OR "man" OR "men" OR "woman" OR "women" OR "adult" OR "adults" OR "middle-age*" OR "elderly")
7. Combine searches	#5 NOT #6
8. Limit results for publication type	#8 has been limited to books & documents<br>#9 has been limited to books & documents, case reports<br>#10 has been limited to books & documents, case reports, comment<br>#11 has been limited books & documents, case reports, comment, editorial<br>#12 has been limited books & documents, case reports, comment, editorial, guideline<br>#13 has been limited books & documents, case reports, comment, editorial, guideline, letter<br>#14 has been limited books & documents, case reports, comment, editorial, guideline, letter, practice guideline<br>#15 has been limited books & documents, case reports, comment, editorial, guideline, letter, practice guideline, preprints<br>16 # #7 NOT #15

Supplementary Table 2: Joanna Briggs Institution Critical Appraisal checklist for cohort and case-control studies

**COHORTS**	Cunning. 2023	Shaffer 2023	Colston 2022	deKoff 2022	Moroishi 2022	Robertson 2021	Parker 2021	Fix 2020	Zhao 2020	Huda 2019	Praharaj 2019	Huda 2014	Mullie 2004	Zhang 2024	Daddi 2023	Ray 2023	Peng 2023	Hirota 2023
1. Groups similar and from **same population**	Y	Y	Y	Y	Y	Y	Y	Y	Y	Y	Y	Y	Y	Y	Y	Y	Y	Y
2. **Exposures** measured **similarly**	Y	Y	Y	Y	Y	Y	Y	Y	Y	Y	Y	Y	Y	Y	Y	Y	Y	Y
3. **Exposure** measurement **valid &reliable**	Y	Y	Y	Y	Y	Y	Y	Y	Y	Y	Y	Y	Y	Y	Y	Y	Y	Y
4. **Confounding** factors identified	Y	Y	Y	N	Y	Y	Y	N	N	Y	N	Y	N	Y	N	Y	Y	Y
5. **Strategies** to address **confounding**	Y	U	Y	N	Y	Y	N	N	N	Y	N	Y	N	Y	Y	Y	Y	Y
6. Participants **free of outcome** at start	Y/N[1]	Y	Y	Y	Y	Y	Y	Y	Y	Y	Y	Y	Y	Y	Y	Y	Y/N[2]	Y
7. **Outcomes** measurement **valid & reliable**	Y	Y	Y	Y	Y	Y	Y	Y	Y	Y	Y	Y	Y	Y	Y	Y	Y	Y
8. **FU** time reported and sufficient	Y	Y	Y	Y	Y	Y	Y	Y	Y	Y	Y	Y	Y	Y	Y	Y	Y	Y
9. **FU complete**, or reasons for LTFU described	Y	N	Y	Y	U	Y	U	Y	Y	U	Y	Y	Y	Y	Y	Y	Y	Y
10. **Strategies** to address **incomplete FU**	Y	N	Y	Y	U	Y	U	Y	Y	U	U	Y	Y	Y	Y	Y	Y	Y
11. Appropriate **statistical analysis**	Y	Y	Y	Y	Y	Y	Y	Y	Y	Y	Y	Y	Y	Y	Y	Y	Y	Y
**PERCENTAGE YES**	100	73	100	82	82	100	73	82	82	82	73	100	82	100	91	100	91	100

**COHORTS**	Ng 2022	Tang 2022	Yuki 2021	Chac 2021	Cait 2021	Shannon 2020	Shortt 2018	Harris 2018	Eloe 2013
1. Groups similar and from **same population**	Y	Y	Y	Y	Y	Y	Y	Y	Y
2. **Exposures** measured **similarly**	Y	Y	Y	Y	Y	Y	Y	Y	Y
3. **Exposure** measurement **valid &reliable**	Y	Y	Y	Y	Y	Y	Y	Y	Y
4. **Confounding** factors identified	Y	U	N	N	N	N	N	N	N
5. **Strategies** to address **confounding**	Y	U	N	N	N	N	N	N	N
6. Participants **free of outcome** at start	Y/N[2]	Y	Y	Y	Y/N[3]	Y	Y/N[3]	N4	U
7. **Outcomes** measurement **valid & reliable**	Y	Y	Y	Y	Y	Y	Y	Y	Y
8. **FU** time reported and sufficient	Y	Y	Y	Y	Y	Y	Y	Y	Y
9. **FU complete**, or reasons for LTFU described	Y	Y	Y	N	Y	Y	Y	Y	Y
10. **Strategies** to address **incomplete FU**	Y	Y	Y	N	N	Y	Y	Y	Y
11. Appropriate **statistical analysis**	Y	Y	Y	Y	U	Y	Y	Y	Y
**PERCENTAGE YES**	91	82	82	64	64	82	82	82	73

**CASE-CONTROLS**	Harris 2018	Parker 2018	Harris 2016
1. **Groups similar** apart from disease/ control	Y	Y	Y
2. Cases and controls **matched appropriately**	Y	N	Y
3. **Same criteria identification** case/control	Y	Y	Y
4. **Exposure** measurement **valid & reliable**	Y	Y	Y
5. **Exposure measured similarly** case/control	Y	Y	Y
6. **Confounding** factors identified	N	N	N
7. **Strategies** to address **confounding** stated	N	N	N
8. **Outcomes** measurement **valid & reliable**	Y	Y	Y
9. **Exposure period sufficient** for outcome	Y	Y	Y
10. Appropriate **statistical analysis**	Y	Y	Y
**PERCENTAGE YES**	80	70	80

Y: yes, N: no, U: unclear. 1: some seropositive at baseline; 2: stools within 3 days of vaccination; 3: only some vaccinated naïve; 4: not RV seronegative, but measuring IgA with 6 days half-life

Supplementary Table 3. Associations between the composition of intestinal microbiome on different taxonomic levels and vaccine responses

Phylum	ACTYNOMYCETOTA[1]	BACILLOTA[2]	PSEUDOMONADOTA[3]	BACTEROIDOTA[4]
	Higher relative abundance associated with<br>• higher polio-specific T cell response, serum IgG [1] and faecal IgA levels[2]<br>• higher tetanus-specific T cell responses and serum IgG levels[1, 3]<br>• higher BCG-specific T cell responses[1, 3]<br>• higher HBV-specific T cell responses[3]	Higher relative abundance associated with<br>• higher rotavirus-specific serum IgA levels[4, 5]	Higher relative abundance associated with<br>• higher rotavirus-specific serum IgA levels[4]	Higher relative abundance associated with<br>• higher SARS-CoV-2-specific plasma IgG levels[6-8]
		Higher relative abundance associated with<br>• low poliovirus-specific faecal IgA[2]<br>• lower tetanus-specific T cell response[1]<br>• lower SARS-CoV-2-specific plasma IgG levels[6-8]	Higher relative abundance associated with<br>• lower BCG-specific T cell and DTH skin-test responses[1]	Higher relative abundance associated with<br>• lower rotavirus-specific IgA serum levels[5]<br>• lower HepB-specific serum IgG levels[9]
**Class**	***Unidentified_Actinobacteria***<br>Higher relative abundance associated with<br>• higher polio-specific T cell response [1] and faecal IgA levels[2]<br>• higher tetanus-specific T cell responses [1]<br>• higher BCG-specific T cell responses[1]	***Clostridia***<br>Higher relative abundance associated with<br>• low poliovirus-specific faecal IgA[2]<br>• lower influenza-specific (H1N1&H3N2) serum IgG levels[10]<br>• lower tetanus-specific T cell response[1]	***Alphaproteobacteria***<br>Higher relative abundance associated with<br>• higher influenza-specific (H1N1) serum IgG levels [10]<br>***Betaproteobacteria***<br>Higher relative abundance associated with<br>• higher BCG-specific T cell responses[1]	
			Betaproteobacteria<br>Higher relative abundance associated with<br>• lower BCG DTH skin-test response[1]<br>***Gammaproteobacteria***<br>Higher relative abundance associated with<br>• lower tetanus-specific T cell responses [1]<br>• lower BCG DTH skin-test response[1]	
**Order**	**Actinomycetales**<br>Higher relative abundance associated with<br>• higher tetanus-specific serum IgG levels[1]<br>• higher BCG-specific T cell and DTH skin-test responses[1]<br>**Bifidobacteriales**<br>Higher relative abundance associated with<br>• higher BCG-specific T cell and DTH skin-test responses[1]<br>• higher tetanus-specific T cell responses[1]<br>• higher polio-specific faecal IgA levels [2]<br>**Coriobacteriales**<br>Higher relative abundance associated with	***Eubacteriales[5]***<br>Higher relative abundance associated with<br>• Higher *V. cholerae*-specific serum IgG levels [11]<br>• higher influenza-specific (H3N2) serum IgG levels[10]	**Burkholderiales**<br>Higher relative abundance associated with<br>• higher BCG-T cell response[1]	
		Eubacteriales[5]<br>Higher relative abundance associated with<br>• lower polio-specific faecal IgA levels [2]<br>• lower tetanus-specific T cell responses[1]<br>• lower HepB-specific serum IgG levels[9]<br>***Bacillales***<br>Higher relative abundance associated with	**Enterobacterales**<br>Higher relative abundance associated with<br>• lower tetanus-specific T cell response[1]<br>• lower BCG-T cell and DTH skin-test responses[1]<br>• lower cholera vaccine cell response [11]<br>**Pseudomonadales**	

	• higher polio-specific T cell response [1]	• lower pneumococcal-specific saliva IgG levels[12]	Higher relative abundance associated with<br>• lower polio-specific T cell response and polio-specific serum IgG levels[1]<br>• lower tetanus-specific T-cell response [1]<br>• lower HepB-specific T-cell response [1]<br>• lower BCG-specific T-cell response [1]	
**Family**	***Coriobacteriaceae***<br>Higher relative abundance associated with<br>• higher polio-specific T cell response[1]<br>***Corynebacteriaceae***<br>Higher relative abundance associated with<br>• higher tetanus-specific T cell response[1]<br>***Bifidobacteriaceae***<br>Higher relative abundance associated with<br>• higher tetanus-specific T cell response[1]<br>• higher BCG-specific T cell and DTH skin-test responses[1]<br>***Micrococcaceae***<br>Higher relative abundance associated with<br>• higher HepB-specific serum IgG levels[1]<br>• higher BCG DTH skin-test response[1]	***Lachnospiraceae***<br>Higher relative abundance associated with<br>• superior *Salmonella* Typhi-specific T cell response[13]<br>• higher influenza-specific (H1N1&H3N2) serum IgG levels)[10]<br>• higher MenC-specific saliva IgG levels[12]<br>• higher HepB-specific serum IgG levels[9]<br>• higher SARS-CoV-2-specific plasma IgG levels[6-8]<br>***Oscillospiraceae***[7]<br>Higher relative abundance associated with<br>• superior *Salmonella* Typhi-specific T cell response [13]<br>• higher influenza-specific (H3N2) serum IgG levels[10]<br>• higher rotavirus-specific serum IgA levels[14]<br>***Peptostreptococcaceae (Clostridium cluster XI)***<br>Higher relative abundance associated with<br>• higher rotavirus-specific IgA levels in serum[4]<br>***Enterococcaceae***<br>Higher relative abundance associated with<br>• higher polio-specific T cell response[1]<br>• higher BCG-specific T cell response[1]<br>***Lactobacillaceae***<br>Higher relative abundance associated with<br>• higher SARS-CoV-2-specific plasma IgG levels[6-8]	***Pseudomonadaceae***<br>Higher relative abundance associated with<br>• higher tetanus-specific T cell response[1]<br>***Enterobacteriaceae***<br>Higher relative abundance associated with<br>• higher pneumococcal-specific saliva IgG levels[12]	***Prevotellaceae***<br>Higher relative abundance associated with<br>• higher rotavirus-specific serum IgA levels[14]<br>***Porphyromonadaceae***<br>Higher relative abundance associated with<br>• higher rotavirus-specific serum IgA levels[14]<br>• higher polio-specific T cell response[1]<br>***Bacteroides***<br>Higher relative abundance associated with<br>• higher pneumococcal-specific saliva IgG levels[12]<br>• higher SARS-CoV-2-specific plasma IgG levels[6-8]<br>***Alistipes***<br>Higher relative abundance associated with<br>• higher SARS-CoV-2-specific plasma IgG levels[15]

	Bifidobacteriaceae<br>Higher relative abundance associated with<br>• lower MenC-specific saliva IgG levels[12]	***Lachnospiraceae***<br>Higher relative abundance associated with<br>• lower influenza-specific (H1N1) serum IgG levels [10]<br>• lower HepB-specific serum IgG levels[1]<br>***Erysipelotrichaceae***<br>Higher relative abundance associated with<br>• higher rotavirus-specific serum IgA levels[14]<br>***Lactobacillaceae***<br>Higher relative abundance associated with<br>• lower tetanus-specific T cell response[1]<br>***Staphylococcaceae***<br>Higher relative abundance associated with<br>• lower pneumococcal-specific saliva IgG levels[12]<br>***Carnobacteriaceae***<br>Higher relative abundance associated with<br>• lower HepB-specific serum IgG levels[1]<br>***Oscillospiraceae***[7]<br>Higher relative abundance associated with<br>• lower SARS-CoV-2-specific plasma IgG levels[6-8]	***Enterobacteriaceae***<br>Higher relative abundance associated with<br>• lower tetanus-specific T cell response[1]<br>• lower pneumococcal-specific saliva IgG levels[12]<br>• lower BCG DTH skin-test response[1]<br>***Moraxellaceae***<br>Higher relative abundance associated with<br>• lower tetanus-specific T cell response[1]<br>• lower BCG-specific T cell response[1]<br>• lower HepB-specific T cell response[1]<br>***Pseudomonadaceae***<br>Higher relative abundance associated with<br>• lower polio-specific T cell response[1]	***Prevotellaceae***<br>Higher relative abundance associated with<br>• lower BCG DTH skin-test response[1]
**Genus**	***Bifidobacterium***<br>Higher relative abundance associated with<br>• higher pneumococcal-specific saliva IgG levels[12]<br>• higher polio-specific serum/plasma IgG and IgA and stool IgA levels[1, 3]<br>• higher tetanus-specific T cell response[1]<br>• higher BCG-specific T cell and DTH skin-test responses[1, 3]<br>• higher HepB-specific T cell responses[3]<br>***Corynebacterium***<br>Higher relative abundance associated with<br>• higher tetanus-specific T cell response [1]<br>***Rothia***<br>Higher relative abundance associated with<br>• higher HepB-specific serum IgG levels[1]<br>• higher BCG-specific serum IgG [1]	***Oscillospira***<br>Higher relative abundance associated with<br>• higher influenza-specific (H1N1) serum IgG levels[10]<br>***Ruminococcus***<br>Higher relative abundance associated with<br>• higher influenza-specific (H3N2) serum IgG levels[10]<br>***Dolosigranulum***<br>Higher relative abundance associated with<br>• higher BCG-specific T cell response[1]<br>***Enterococcus***<br>Higher relative abundance associated with<br>• higher BCG-specific T cell response[1]<br>***Blautia***<br>Higher relative abundance associated with<br>• higher pneumococcal-specific saliva IgG levels[12]<br>***Subdoligranulum***	***Serratia***<br>Higher relative abundance associated with<br>• higher rotavirus-specific IgA levels in serum[4]<br>***Sutterella***<br>Higher relative abundance associated with<br>• higher influenza-specific (H1N1) serum IgG levels[10]<br>• higher SARS-CoV-2-specific plasma IgG levels[6-8]<br>***Esch/Shigella***<br>Higher relative abundance associated with<br>• higher pneumococcal-specific saliva IgG levels[12]	***Bacteroides***<br>Higher relative abundance associated with<br>• lower rotavirus-specific IgA serum levels[5]<br>• lower HepB-specific serum IgG levels[9]<br>• lower *V. cholerae*-specific serum IgG levels [16]<br>• higher SARS-CoV-2-specific plasma IgG levels[15]<br>***Prevotella***<br>Higher relative abundance associated with<br>• lower rotavirus-specific serum IgA levels[5]<br>• lower pneumococcal-specific saliva IgG levels[2]<br>• lower BCG DTH skin-test response[1]<br>***Butyricimonas***

		Higher relative abundance associated with<br>• higher pneumococcal-specific saliva IgG levels[12]<br>***Roseburia***<br>Higher relative abundance associated with<br>• higher MenC-specific saliva IgG levels[12]<br>***Lachnospira***<br>Higher relative abundance associated with<br>• higher MenC-specific saliva IgG levels[12]<br>***Pseudobutyrivibrio***<br>Higher relative abundance associated with<br>• higher MenC-specific saliva IgG levels[12]<br>***Butyricoccus***<br>Higher relative abundance associated with<br>• higher HepB-specific serum IgG levels[9]<br>***Phascolarctobacterium***<br>Higher relative abundance associated with<br>• higher HepB-specific serum IgG levels[9]<br>***Clostridium***<br>Higher relative abundance associated with<br>• higher HepB-specific serum IgG levels[9]<br>***Solobacterium***<br>Higher relative abundance associated with<br>• higher SARS-CoV-2-specific plasma IgG levels[6, 7]<br>***Lactobacillus***<br>Higher relative abundance associated with<br>• lower SARS-CoV-2-specific plasma IgG levels[6-8]		Higher relative abundance associated with<br>• lower influenza-specific (H1N1) serum IgG levels[10]<br>***Alloprevotella***<br>Higher relative abundance associated with<br>• lower SARS-CoV-2-specific plasma IgG levels[6-8]
	Gardnerella<br>Higher relative abundance associated with<br>• lower pneumococcal-specific saliva IgG levels[12]<br>• lower HepB-specific serum IgG levels[9]<br>***Atopobium***<br>Higher relative abundance associated with<br>• lower HepB-specific serum IgG levels[9]	***Phascolarctobacterium***<br>Higher relative abundance associated with<br>• lower influenza-specific (H1N) serum IgG levels[10]<br>***Lactococcus***<br>Higher relative abundance associated with<br>• lower BCG-specific T cell response[1]<br>• lower polio-specific T cell response[1]<br>• lower pneumococcal-specific saliva IgG levels[12]<br>***Lactobacillus***<br>Higher relative abundance associated with<br>• lower HepB-specific T cell response[1]	***Acinetobacter***<br>Higher relative abundance associated with<br>• lower BCG-specific T cell response[1]<br>• lower tetanus-specific T cell response[1]<br>***Esch/Shigella***<br>Higher relative abundance associated with<br>• lower tetanus-specific T cell response[1]<br>***Klebsiella***<br>Higher relative abundance associated with<br>• lower pneumococcal-specific saliva IgG levels[2]<br>• lower MenC-specific saliva IgG levels[12]<br>***Succinivibrio***	

		Enterococcus<br>Higher relative abundance associated with<br>• lower pneumococcal-specific saliva IgG levels[12]<br>***Blautia***<br>Higher relative abundance associated with<br>• lower HepB-specific serum IgG levels[9]<br>***Dialister***<br>Higher relative abundance associated with<br>• lower HepB-specific serum IgG levels[9]<br>***Megasphaera***<br>Higher relative abundance associated with<br>• lower HepB-specific serum IgG levels[9]<br>***Clostridium sensu stricto***<br>Higher relative abundance associated with<br>• lower polio-specific faecal IgA levels [2]<br>• lower pneumococcal-specific saliva IgG levels[12]<br>***Turicibacter***<br>Higher relative abundance associated with<br>• lower influenza-specific (H1N1&H3N2) serum IgG levels[10]<br>***Clostridium***<br>Higher relative abundance associated with<br>• lower influenza-specific (H1N1&H3N2) serum IgG levels[10]<br>***Oscillospira (Clostridia)***<br>Higher relative abundance associated with<br>• lower influenza-specific (H1N1) serum IgG levels[10]<br>***Coprococcus***<br>Higher relative abundance associated with<br>• lower influenza-specific (H3N2) serum IgG levels[10]<br>***Streptococcus***<br>Higher relative abundance associated with<br>• lower influenza-specific (H3N2) serum IgG levels[10]<br>• lower pneumococcal-specific saliva IgG levels[12]<br>***Veillonella***<br>Higher relative abundance associated with<br>• lower MenC-specific saliva IgG levels[12]	Higher relative abundance associated with<br>• lower SARS-CoV-2-specific plasma IgG levels[6-8]	

		• lower pneumococcal-specific saliva IgG levels[2]<br>***Clostridium XI, Finegoldia, Peptoniphilus, Megasphaera***<br>Higher relative abundance associated with<br>• lower BCG DTH skin-test response[1]<br>***Colidextribacter***<br>Higher relative abundance associated with<br>• lower SARS-CoV-2-specific plasma IgG levels[15]<br>***Lachnoclostridium***<br>Higher relative abundance associated with<br>• lower SARS-CoV-2-specific plasma IgG levels[15]<br>***Anaerofilum, Ruminiclostridium, Hydrogenoanaerobacterium***<br>Higher relative abundance associated with<br>• lower SARS-CoV-2-specific plasma IgG levels[6-8]<br>***Moryella, Marvinbryantia***<br>Higher relative abundance associated with<br>• lower SARS-CoV-2-specific plasma IgG levels[6-8]		
**Species**	***B. longum***<br>Higher relative abundance associated with<br>• higher tetanus-specific T cell responses and serum IgG levels[3]<br>• higher polio-specific plasma IgA[3], serum IgG and T cell response[1]<br>• higher BCG-specific T cell responses[3]<br>• higher HepB-specific T cell responses[3]<br>***B. longum* subspecies *infantis***<br>Higher relative abundance associated with<br>• higher polio-specific T cell response and serum IgG levels[1]<br>• higher BCG-specific T cell responses[1, 3]<br>• higher tetanus-specific T cell responses[1, 3]<br>Detectable levels associated with<br>• higher polio-specific faecal IgA levels[17]<br>***B. longum* subspecies *longum***<br>Higher relative abundance associated with<br>• higher polio-specific T cell response[1]<br>***B. adolescentis***<br>Higher relative abundance associated with<br>• higher SARS-CoV-2-specific plasma IgG levels[6, 7]	***Streptococcus bovis***<br>Higher relative abundance associated with<br>• higher rotavirus-specific serum IgA levels[5]<br>• higher pneumococcal-specific saliva IgG levels[12]<br>***Streptococcus gallolyticus***<br>Higher relative abundance associated with<br>• higher pneumococcal-specific saliva IgG levels[12]<br>***Roseburia faecis***<br>Higher relative abundance associated with<br>• higher SARS-CoV-2-specific plasma IgG levels [6, 7]<br>***Roseburia intestinalis***<br>Higher relative abundance associated with<br>• higher SARS-CoV-2-specific plasma IgG levels[6, 7]<br>***Agathobacter rectalis*[6]**<br>Higher relative abundance associated with<br>• higher SARS-CoV-2-specific plasma IgG levels [6]<br>***Eubacterium ramulus***<br>Higher relative abundance associated with	***E. coli***<br>Higher relative abundance associated with<br>• higher rotavirus-specific serum IgA levels[4]<br>• higher pneumococcal-specific saliva IgG levels[12]<br>***Shigella dysentariae, sonnei & flexneri***<br>Higher relative abundance associated with<br>• Higher *V. cholerae*-specific serum IgG levels [16]<br>***Parasutterella excrementihominis***<br>Higher relative abundance associated with<br>• higher SARS-CoV-2-specific plasma IgG levels[6, 7]	***Bacteroides thetaiotaomicron***<br>Higher relative abundance associated with<br>• higher rotavirus-specific serum IgA levels[19]<br>• higher SARS-CoV-2-specific plasma IgG levels[6]<br>***Parabacteroides distasonis***<br>Higher relative abundance associated with<br>• higher influenza-specific (H1N1) serum IgG levels[10]<br>***Parabacteroides merdae***<br>Higher relative abundance associated with<br>• higher rotavirus-specific serum IgA levels[14]<br>***Prevotella copri***<br>Higher relative abundance associated with<br>• higher influenza-specific (H3N2) serum IgG levels[10]

	B. bifidum<br>Higher relative abundance associated with<br>• higher influenza-specific (H3N2) serum IgG levels[10]<br>• higher polio-specific plasma IgG[3]<br>• higher SARS-CoV-2-specific plasma IgG levels[6, 7]<br><br>***Collinsella aerofaciens***<br>Higher relative abundance associated with<br>• higher SARS-COV2-specific serum IgG levels[18]	• higher SARS-COV2-specific serum IgG levels[18]<br><br>***Faecalibacterium prausnitzii***<br>Higher relative abundance associated with<br>• higher rotavirus-specific serum IgA levels[14]<br>• higher pneumococcal-specific saliva IgG levels[12]<br><br>***Ruminococcus gnavus***<br>Higher relative abundance associated with<br>• higher pneumococcal-specific saliva IgG levels[12]<br><br>***Oscillospira (Clostridia)***<br>Higher relative abundance associated with<br>• higher influenza-specific (H1N1) serum IgG levels[10]<br><br>***Veillonella dispar***<br>Higher relative abundance associated with<br>• higher SARS-COV2-specific serum IgG levels[18]<br><br>***Bacillus licheniformis***<br>Higher relative abundance associated with<br>• Higher *V. cholerae*-specific serum IgG levels [16]<br><br>***Lachnospira pectinoschizia***<br>Higher relative abundance associated with<br>• higher SARS-CoV-2-specific plasma IgG levels[6, 7]<br><br>***Lactococcus lactis***<br>Higher relative abundance associated with<br>• higher SARS-CoV-2-specific plasma IgG levels[6, 7]<br><br>***Dorea formicigenerans***<br>Higher relative abundance associated with<br>• higher SARS-CoV-2-specific plasma IgG levels[6, 7]<br><br>***Coprococcus catus***<br>Higher relative abundance associated with<br>• higher SARS-CoV-2-specific plasma IgG levels[6, 7]<br><br>***Blautia massiliensis***<br>Higher relative abundance associated with<br>• higher SARS-CoV-2-specific plasma IgG levels[6, 7]		• higher rotavirus-specific serum IgA levels[14]<br><br>***Phocaeicola dorei***<br>Higher relative abundance associated with<br>• higher SARS-CoV-2-specific plasma IgG levels[6, 7]<br><br>***Rikenellaceae bacterium***<br>Higher relative abundance associated with<br>• higher SARS-CoV-2-specific plasma IgG levels[6, 7]
	B. infantis	***Ruminococcus gnavus***	***Haemophilus parainfluenzae***	***Bacteroides vulgatus***

	Higher relative abundance associated with<br>• lower polio-specific serum IgG[20]<br>***B. breve***<br>Higher relative abundance associated with<br>• lower HepB-specific serum IgG levels[3]<br>• lower polio-specific plasma IgA[3]<br>***B. longum* subspecies *longum***<br>Higher relative abundance associated with<br>• lower HepB-specific serum IgG levels[3]<br>• lower polio-specific plasma IgG[3]<br>***B. bifidum***<br>Higher relative abundance associated with<br>• lower pneumococcal-specific saliva IgG levels[12]<br>***B. animalis***<br>Higher relative abundance associated with<br>• lower pneumococcal-specific saliva IgG levels[12]<br>***Aeriscardovia aeriphila***<br>Higher relative abundance associated with<br>• lower tetanus-toxoid-specific serum IgG levels[21]<br>***Corynebacterium propinquum***<br>Higher relative abundance associated with<br>• lower pneumococcal-specific saliva IgG levels[12]<br>***Colinsella aerofaciens***<br>Higher relative abundance associated with<br>• higher SARS-CoV-2-specific plasma IgG levels[22]	Higher relative abundance associated with<br>• lower SARS-CoV-2-specific plasma IgG levels[6]<br>***Clostridium_XVIII ramosum***<br>Higher relative abundance associated with<br>• lower rotavirus-specific serum IgA levels[14]<br>• lower pneumococcal-specific saliva IgG levels[12]<br>***Streptococcus pyogenes***<br>Higher relative abundance associated with<br>• lower pneumococcal-specific saliva IgG levels[12]<br>***Streptococcus salivarius***<br>Higher relative abundance associated with<br>• lower SARS-CoV-2-specific plasma IgG levels[22]<br>***Lawsonibacter asaccharolyticus***<br>Higher relative abundance associated with<br>• higher SARS-COV2-specific serum IgG levels[18]<br>***Agathobacter rectalis***[6]<br>Higher relative abundance associated with<br>• higher SARS-CoV-2-specific plasma IgG levels[22]<br>***Clostridium innocuum***<br>Higher relative abundance associated with<br>• lower SARS-CoV-2-specific plasma IgG levels[15]<br>***Latilactobacillus sakei***<br>Higher relative abundance associated with<br>• lower SARS-CoV-2-specific plasma IgG levels[6, 7]<br>***Emergencia timonensis***<br>Higher relative abundance associated with<br>• lower SARS-CoV-2-specific plasma IgG levels[6, 7]<br>***Lacrimispora saccharolytica***<br>Higher relative abundance associated with<br>• lower SARS-CoV-2-specific plasma IgG levels[6, 7]<br>***Parabacteroides faecis***<br>Higher relative abundance associated with	Higher relative abundance associated with<br>• lower influenza-specific (H3N2) serum IgG levels [10]<br>***Citrobacter sedlakii***<br>Higher relative abundance associated with<br>• lower pneumococcal-specific saliva IgG levels[2]<br>***Citrobacter freundii***<br>Higher relative abundance associated with<br>• lower SARS-CoV-2-specific plasma IgG levels[6, 7]	Higher relative abundance associated with<br>• lower SARS-CoV-2-specific plasma IgG levels[6]<br>***Bacteroides thetaiotaomicron***<br>Higher relative abundance associated with<br>• lower SARS-CoV-2-specific plasma IgG levels[6]<br>***Bacteroides ovatus***<br>Higher relative abundance associated with<br>• lower SARS-CoV-2-specific plasma IgG levels[6, 7]<br>***Alistipes dispar***<br>Higher relative abundance associated with<br>• higher SARS-CoV-2-specific plasma IgG levels[6, 7]<br>***Parabacteroides faecis***<br>Higher relative abundance associated with<br>• lower SARS-CoV-2-specific plasma IgG levels[6, 7]<br>***Paraprevotella clara***<br>Higher relative abundance associated with<br>• lower SARS-CoV-2-specific plasma IgG levels[6, 7]<br>***Parabacteroides johnsonii***<br>Higher relative abundance associated with<br>• lower SARS-CoV-2-specific plasma IgG levels[6, 7]

		• lower SARS-CoV-2-specific plasma IgG levels[6, 7]<br><br>***Intestinimonas butyriciproducens***<br>Higher relative abundance associated with<br>• lower SARS-CoV-2-specific plasma IgG levels[6, 7]<br><br>***Enterocloster citroniae, bolteae & lavalensis***<br>Higher relative abundance associated with<br>• lower SARS-CoV-2-specific plasma IgG levels[6, 7]<br><br>***Dysosmobacter welbionis***<br>Higher relative abundance associated with<br>• lower SARS-CoV-2-specific plasma IgG levels[6, 7]<br><br>***Clostridium fessum***<br>Higher relative abundance associated with<br>• lower SARS-CoV-2-specific plasma IgG levels[6, 7]<br><br>***Faecalibacterium prausnitzii***<br>Higher relative abundance associated with<br>• lower SARS-CoV-2-specific plasma IgG levels[6, 7]		

Green - relative abundance associated with higher vaccine responses; red - relative abundance associated with lower vaccine responses

Other: cyanobacteria (Phylum) associated with higher vaccine response H3N2)[10], *Slackia isoflavoniconvertens* associated with higher rotavirus-specific serum IgA levels ($p<0.05$)[19]; Erysipelotrichaceae (family), Fusobacteriaceae (family) and *Fusobacterium* (genus) associated with lower serum anti-RV IgA [14] and lower HepB-specific serum IgG levels[9], *Bilophila wadsworthia* associated with higher pneumococcal-specific saliva IgG levels[2]; Desulfobacterota and *Bilophila* associated with higher SARS-CoV-2-specific plasma IgG levels[15]; Desulfobivrionales associated with lower relative abundance of HepB-specific serum IgG levels[9], *Anaerobaculum mobile* associated with higher *V. cholerae*-specific serum IgG levels [16], *Akkermansia* and *Akkermansiaceae* (phylum Verrumicrobiota) associated with lower SARS-CoV-2-specific plasma IgG levels[6-8]. All ($p<0.05$).

HepB – hepatitis B virus; MenC – meningococcus C;

1: Previous Actinobacteria; 2: Previous Firmicutes; 3: Previous Proteobacteria; 4: Previous Bacteroidetes; 5: Previous/other: clostridiales; 6: Previous *Eubacterium rectale,* 7: Previous *Ruminocaccaceae*

References

1. Huda, M.N., et al., *Stool microbiota and vaccine responses of infants.* Pediatrics, 2014. **134**(2): p. e362-72.
2. Zhao, T., et al., *Influence of gut microbiota on mucosal IgA antibody response to the polio vaccine.* NPJ Vaccines, 2020. **5**(1): p. 47.
3. Huda, M.N., et al., *Bifidobacterium Abundance in Early Infancy and Vaccine Response at 2 Years of Age.* Pediatrics, 2019. **143**(2).
4. Harris, V., et al., *Rotavirus vaccine response correlates with the infant gut microbiota composition in Pakistan.* Gut Microbes, 2018. **9**(2): p. 93-101.
5. Harris, V.C., et al., *The infant gut microbiome correlates significantly with rotavirus vaccine response in rural Ghana.* J Infect Dis, 2016.
6. Ng, S.C., et al., *Gut microbiota composition is associated with SARS-CoV-2 vaccine immunogenicity and adverse events.* Gut, 2022. **71**(6): p. 1106-1116.
7. Peng, Y., et al., *Baseline gut microbiota and metabolome predict durable immunogenicity to SARS-CoV-2 vaccines.* Signal Transduction and Targeted Therapy, 2023. **8**(1): p. 373.
8. Ray, S., et al., *Impact of the gut microbiome on immunological responses to COVID-19 vaccination in healthy controls and people living with HIV.* npj Biofilms and Microbiomes, 2023. **9**(1): p. 104.
9. Shannon, C.P., et al., *Multi-omic data integration allows baseline immune signatures to predict hepatitis B vaccine response in a small cohort.* Frontiers in immunology, 2020. **11**: p. 578801.
10. Cait, A., et al., *Potential association between dietary fibre and humoral response to the seasonal influenza vaccine.* Frontiers in Immunology, 2021. **12**: p. 765528.
11. Chac, D., et al., *Gut Microbiota and Development of Vibrio cholerae-Specific Long-Term Memory B Cells in Adults after Whole-Cell Killed Oral Cholera Vaccine.* Infect Immun, 2021. **89**(9): p. e0021721.
12. de Koff, E.M., et al., *Mode of delivery modulates the intestinal microbiota and impacts the response to vaccination.* Nature Communications, 2022. **13**(1): p. 6638.
13. Eloe-Fadrosh, E.A., et al., *Impact of oral typhoid vaccination on the human gut microbiota and correlations with s. Typhi-specific immunological responses.* PLoS One, 2013. **8**(4): p. e62026.
14. Harris, V.C., et al., *Effect of antibiotic-mediated microbiome modulation on rotavirus vaccine immunogenicity: a human, randomized-control proof-of-concept trial.* Cell host & microbe, 2018. **24**(2): p. 197-207. e4.
15. Daddi, L., et al., *Baseline Gut Microbiome Signatures Correlate with Immunogenicity of SARS-CoV-2 mRNA Vaccines.* International Journal of Molecular Sciences, 2023. **24**(14): p. 11703.
16. Yuki, Y., et al., *Oral MucoRice-CTB vaccine for safety and microbiota-dependent immunogenicity in humans: a phase 1 randomised trial.* The Lancet Microbe, 2021. **2**(9): p. e429-e440.
17. Mullié, C., et al., *Increased poliovirus-specific intestinal antibody response coincides with promotion of Bifidobacterium longum-infantis and Bifidobacterium breve in infants: a randomized, double-blind, placebo-controlled trial.* Pediatr Res, 2004. **56**(5): p. 791-5.
18. Tang, B., et al., *Correlation of gut microbiota and metabolic functions with the antibody response to the BBIBP-CorV vaccine.* Cell Reports Medicine, 2022. **3**(10).
19. Robertson, R.C., et al., *The fecal microbiome and rotavirus vaccine immunogenicity in rural Zimbabwean infants.* Vaccine, 2021. **39**(38): p. 5391-5400.
20. Colston, J.M., et al., *Intestinal colonization with Bifidobacterium longum subspecies is associated with length at birth, exclusive breastfeeding, and decreased risk of enteric virus infections, but not with histo-blood group antigens, oral vaccine response or later growth in three birth cohorts.* Frontiers in Pediatrics, 2022. **10**: p. 804798.
21. Moroishi, Y., et al., *A prospective study of the infant gut microbiome in relation to vaccine response.* Pediatric research, 2023. **93**(3): p. 725-731.
22. Zhang, L.-N., et al., *Association between Gut Microbiota Composition and Long-Term Vaccine Immunogenicity following Three Doses of CoronaVac.* Vaccines, 2024. **12**(4): p. 365.

Supplementary Table 4 Associations between the composition of the intestinal microbiota at different taxonomic levels and vaccine responses in **infants** (colour coding: higher relative abundance associated with higher vaccine response (blue); lower vaccine response (yellow), contradictory findings (grey); all p<0.05)

	Oral		Parenteral		
**Phylum**	OPV	ORV	BCG	TT	HBV
**Actynomycetota[1]**					
**Bacillota[2]**					

	Oral	Parenteral	
**Class**	OPV	BCG	TT
Actinobacteria			
Coriobacteriia			
Bacilli			
Tissierellia			
Clostridia			

	Oral	Parenteral			
**Order**	OPV	BCG	TT	HBV	PCV
Actinomycetales					
Bifidobacteriales					
Coriobacteriales					
Bacillales					
Lactobacillales					
Tissierellales					
Lachnospirales					
Eubacteriales[7]					
Peptostreptococcales					

	Oral		Parenteral				
**Family**	OPV	ORV	BCG	TT	HBV	MenC	PCV
Actinomycetaceae							
Corynebacteriaceae							
Micrococcaceae							
Bifidobacteriaceae							
Coriobacteriaceae							
Staphylococcaceae							
Carnobacteriaceae							
Enterococcaceae							
Lactobacillaceae							
Streptococcaceae							
Peptoniphilaceae							
Lachnospiraceae							
Oscillospiraceae[8]							
Butyricicoccaceae							
Clostridiaceae							
Peptostreptococcaceae							

	Oral		Parenteral				
**Genus/ Species**	OPV	ORV	BCG	TT	HBV	MenC	PCV
Actinomyces							
Corynebacterium							
C. propinquum							
Rothia							
Bifidobacterium							
B. longum							
B. l. infantis							
B. l. longum							
B. bifidum							
B. breve							
Aeriscardovia aeriphila							
Garnerella							
Staphylococcus							
Dolosigranulum							
Enterococcus							
Lactobacillus							
Lactococcus							
Streptococcus							
S. pyogenes							
S. bovis							
Finegoldia							
Peptoniphilus							
Roseburia							
Lachnospira							
Pseudobutyrivibrio							
Blautia							
Subdoligranulum							
Faecalibacterium prausnitzii							
Lawsonibacter asaccharolyticus							
Ruminococcus gnavus							
Butyricoccus							
Clostridium							
Clostridium XVIII							

						Negativicutes				Veillonellales						*Veillonellaceae*								*Megaesphaera*							
																								Veillonella							
**Pseudomo-nadota[3]**						βProteobacteria				Burkholderiales																					
						γProteobac-teria				Enterobacte-rales						*Enterobacteriaceae*								*Escherichia/Shigella*							
																								E. coli							
																								S. dysentariae							
																								S. sonnei							
																								S. flexneri							
																								Citrobacter sedlakii							
																								Klebsiella							
																Yersiniaceae								*Serratia*							
										Pseudomona-dales						*Moraxellaceae*								*Acinetobacter*							
																Pseudomonadaceae															
										Pasteurellales						*Pasteurellaceae*								*H. parainfluenzae*							
**Bacteroidota[4]**						Bacteroidia				Bacteroidales						*Bacteroidaceae*								*Bacteroides*							
																								B. thetaiotaom							
																Porphyromonadaceae								*Parabacteroides merdae*							
																Prevotellaceae								*Prevotella (copri)*							
**Fusobacte-riota[5]**						Fusobacteriia				Fusobacteriales						*Fusobacteriaceae*								*Fusobacterium*							
**Phylum**	**OPV**	**ORV**	**BCG**	**TT**	**HBV**	**Class**	**OPV**	**BCG**	**TT**	**Order**	**OPV**	**BCG**	**TT**	**HBV**	**PCV**	**Family**	**OPV**	**ORV**	**BCG**	**TT**	**HBV**	**MenC**	**PCV**	**Genus/ Species**	**OPV**	**ORV**	**BCG**	**TT**	**HBV**	**MenC**	**PCV**

BCG - Bacille-Calmette-Guérin vaccine; COVID - SARS-CoV-2 vaccine; HBV - HepB vaccine; MenC - meningococcus C polysaccharide vaccine; OCV - oral cholera vaccine; OPV - oral poliovirus vaccine; ORV - oral rotavirus vaccine; PCV - pneumococcal conjugate vaccine, *S. typhi*: oral *Salmonella typhi* vaccine; TIV - trivalent influenza vaccine; TT - tetanus vaccine
1: Previous Actinobacteria; 2: Previous Firmicutes; 3: Previous Proteobacteria; 4: Previous Bacteroidetes; 5: Previous Fusobacteria; 6: Previous Eubacterium rectale; 7: Previous/other: clostridiales; 8: Previous Ruminocaccaceae

Supplementary Table 5 Associations between the composition of the intestinal microbiota at different taxonomic levels and vaccine responses **in adults** (colour coding: higher relative abundance associated with higher vaccine response (blue); lower vaccine response (yellow), contradictory findings (grey); all p<0.05)

Phylum	ORV	HBV	COVID	Class	COVID	TIV	Order	OCV	HBV	COVID	TIV	Family	ORV	*S. typhi*	HBV	COVID	TIV	Genus/ Species	ORV	OCV	HBV	COVID	TIV
**Actynomycetota**[1]				Actinobacteria			Actinomycetales					*Actinomycetaceae*						*Actinomyces*				blue	
							Bifidobacteriales					*Bifidobacteriaceae*						*Bifidobacterium adolescentis*				blue	
																		B. bifidum				blue	blue
																		Garnerella			yellow		
				Coriobacteriia			Coriobacteriales					*Coriobacteriaceae*						*Collinsella aerofaciens*				grey	
												Atopobiaceae						*Atopobium*			yellow		
**Bacillota**[2]	blue		yellow	Bacilli			Bacillales					*Bacillaceae*						*Bacillus licheniformis*		blue			
							Lactobacillales					*Lactobacillaceae*				blue		*Lactobacillus*				blue	
																		Latilactobacillus sakei				yellow	
												Streptococcaceae						*Lactococcus lactis*				blue	
																		Streptococcus					yellow
																		S. salivarius				yellow	
																		S. bovis	blue				
				Clostridia		yellow	Lachnospirales					*Lachnospiraceae*		blue	grey	blue	grey	*Roseburia*				yellow	
																		R. intestinalis				blue	
																		R. faecis				blue	
																		Lachnospira pectinoschizia				blue	
																		Lachnoclostridium				yellow	
																		Lacrimispora Saccharolytica				yellow	
																		Marvinbryantia				yellow	
																		Moryella				yellow	
																		Agathobacter rectalis[6]				blue	
																		Dorea formicigenerans				blue	
																		Blautia			yellow	blue	
																		B. massiliensis				blue	
																		Fusicatenibacter saccharivorans				blue	
																		Enterocloster citroniae				yellow	
																		Enterocloster bolteae				yellow	
																		Enterocloster lavalensis				yellow	
																		Coprococcus					yellow
																		C. catus				blue	
							Eubacteriales[7]	blue	yellow		blue	*Oscillospiraceae*[8]		blue		yellow	blue	*Oscillospira*					grey
																		Anaerofilum				yellow	
																		Dysosmobacter welbionis				yellow	
																		Faecalibacterium prausnitzii	blue			yellow	
																		Ruminiclostridium				yellow	
																		Hydrogenoanaerobacterium				yellow	
																		Ruminococcus					blue
																		R. gnavus				yellow	
												Butyricicoccaceae						*Butyricoccus*			blue		

												Clostridiaceae						*Clostridium*					
																		Clostridium fessum					
												Eubacteriaceae						*Eubacterium ramulus*					
																		Eubacterium limosum					
							Peptostreptococcales					*Anaerovoracaceae*						*Emergencia timonensis*					
												Peptostreptococcaceae											
				Negativicutes			Acidaminococcales					*Acidaminococcaceae*						*Phascolarctobacterium*					
							Veillonellales					*Veillonellaceae*						*Veillonella dispar*					
																		Phascolarcobacterium					
																		Dialister					
				Erysipelotrichia			Erysipelotrichales					*Turicibacteraceae*						*Turicibacter*					
												Erysipelotrichaceae						*Solobacterium*					
																		Clostridium innocuum					
**Pseudomonadota[3]**				βProteobacteria			Burkholderiales					*Sutterelaceae*						*Sutterella*					
																		Parasutterella excrementihomini					
				γProteobac-teria			Enterobacterales					*Enterobacteriaceae*						*Escherichia coli*					
																		Shigella dysentariae					
																		S. sonnei					
																		S. flexneri					
												Yersiniaceae						*Serratia*					
							Aeromonadales					*Succinivibrionaceae*						*Succinivibrio*					
							Pseudomonadales					*Moraxellaceae*											
							Pasteurellales					*Pasteurellaceae*						*H. parainfluenzae*					
				αProteobacteria																			
**Bacteroidota[4]**				Bacteroidia			Bacteroidales					*Bacteroidaceae*						*Bacteroides*					
																		B. ovatus					
																		B. vulgatus					
																		B. thetaiotaom					
																		Phocaeicola dorei					
																		Butyricimonas					
												Tannerellaceae						*Parabacteroides faecis*					
												Rikenellaceae						*Alistipes*					
																		A. dispar					
												Porphyromonadaceae						*Parabacteroides merdae*					
																		Parabacteroides distasonis					
												Prevotellaceae						*Alloprevotella*					
																		Prevotella (copri)					
**Fusobacteriota[5]**				Fusobacteriia			Fusobacteriales					*Fusobacteriaceae*						*Fusobacterium*					
																		F. ulcerans					
**Synergistota**				Synergistia			Synergistales					*Acetomicrobiaceae*						*Anaerobaculum mobile*					
**Thermodesulfobacteriota**				Desulfovibrionia			Desulfovibrionales					*Desulfovibrionaceae*						*Bilophila*					
**Verrucomicrobiota**				Verrucomicrobiia			Verrucomicrobiales					*Akkermansiaceae*						*Akkermansia*					
**Phylum**	ORV	HBV	COVID	**Class**	COVID	TIV	**Order**	OCV	HBV	COVID	TIV	**Family**	ORV	*S. typhi*	HBV	COVID	TIV	**Genus/ Species**	ORV	OCV	HBV	COVID	TIV

BCG - Bacille-Calmette-Guérin vaccine; COVID - SARS-CoV-2 vaccine; HBV - HepB vaccine; MenC - meningococcus C polysaccharide vaccine; OCV - oral cholera vaccine; OPV - oral poliovirus vaccine; ORV - oral rotavirus vaccine; PCV - pneumococcal conjugate vaccine, *S. typhi*: oral *Salmonella typhi* vaccine; TIV - trivalent influenza vaccine; TT - tetanus vaccine
1: Previous Actinobacteria; 2: Previous Firmicutes; 3: Previous Proteobacteria; 4: Previous Bacteroidetes; 5: Previous Fusobacteria; 6: Previous Eubacterium rectale; 7: Previous/other: clostridiales; 8: Previous Ruminocaccaceae

Supplementary Table 6: PRISMA 2020 Main Checklist

Topic	No.	Item	Location where item is reported
**TITLE**			
**Title**	1	Identify the report as a systematic review.	Page1
**ABSTRACT**			
**Abstract**	2	See the PRISMA 2020 for Abstracts checklist	
**INTRODUCTION**			
**Rationale**	3	Describe the rationale for the review in the context of existing knowledge.	Page 3
**Objectives**	4	Provide an explicit statement of the objective(s) or question(s) the review addresses.	Page 4
**METHODS**			
**Eligibility criteria**	5	Specify the inclusion and exclusion criteria for the review and how studies were grouped for the syntheses.	Page 19
**Information sources**	6	Specify all databases, registers, websites, organisations, reference lists and other sources searched or consulted to identify studies. Specify the date when each source was last searched or consulted.	Page 19
**Search strategy**	7	Present the full search strategies for all databases, registers and websites, including any filters and limits used.	Page 19 & Supplementary Table 1
**Selection process**	8	Specify the methods used to decide whether a study met the inclusion criteria of the review, including how many reviewers screened each record and each report retrieved, whether they worked independently, and if applicable, details of automation tools used in the process.	Page 20
**Data collection process**	9	Specify the methods used to collect data from reports, including how many reviewers collected data from each report, whether they worked independently, any processes for obtaining or confirming data from study investigators, and if applicable, details of automation tools used in the process.	Page 20
**Data items**	10a	List and define all outcomes for which data were sought. Specify whether all results that were compatible with each outcome domain in each study were sought (e.g. for all measures, time points, analyses), and if not, the methods used to decide which results to collect.	Page 20 and Table 1

Topic	No.	Item	Location where item is reported
	10b	List and define all other variables for which data were sought (e.g. participant and intervention characteristics, funding sources). Describe any assumptions made about any missing or unclear information.	Page 20 and Table 1
**Study risk of bias assessment**	11	Specify the methods used to assess risk of bias in the included studies, including details of the tool(s) used, how many reviewers assessed each study and whether they worked independently, and if applicable, details of automation tools used in the process.	Page 20
**Effect measures**	12	Specify for each outcome the effect measure(s) (e.g. risk ratio, mean difference) used in the synthesis or presentation of results.	NA
**Synthesis methods**	13a	Describe the processes used to decide which studies were eligible for each synthesis (e.g. tabulating the study intervention characteristics and comparing against the planned groups for each synthesis (item 5)).	Page 20
	13b	Describe any methods required to prepare the data for presentation or synthesis, such as handling of missing summary statistics, or data conversions.	NA
	13c	Describe any methods used to tabulate or visually display results of individual studies and syntheses.	Page 20
	13d	Describe any methods used to synthesize results and provide a rationale for the choice(s). If meta-analysis was performed, describe the model(s), method(s) to identify the presence and extent of statistical heterogeneity, and software package(s) used.	Page 20
	13e	Describe any methods used to explore possible causes of heterogeneity among study results (e.g. subgroup analysis, meta-regression).	NA
	13f	Describe any sensitivity analyses conducted to assess robustness of the synthesized results.	NA
**Reporting bias assessment**	14	Describe any methods used to assess risk of bias due to missing results in a synthesis (arising from reporting biases).	-
**Certainty assessment**	15	Describe any methods used to assess certainty (or confidence) in the body of evidence for an outcome.	Page 20
**RESULTS**			
**Study selection**	16a	Describe the results of the search and selection process, from the number of records identified in the search to the number of studies included in the review, ideally using a flow diagram.	Page 4 & Figure 1
	16b	Cite studies that might appear to meet the inclusion criteria, but which were excluded, and explain why they were excluded.	Figure 1
**Study characteristics**	17	Cite each included study and present its characteristics.	Page 4-5 and Table 1

Topic	No.	Item	Location where item is reported
**Risk of bias in studies**	18	Present assessments of risk of bias for each included study.	Page 5 & Supplementary Table 2
**Results of individual studies**	19	For all outcomes, present, for each study: (a) summary statistics for each group (where appropriate) and (b) an effect estimate and its precision (e.g. confidence/credible interval), ideally using structured tables or plots.	Pages 6-13 & Tables 1-2
**Results of syntheses**	20a	For each synthesis, briefly summarise the characteristics and risk of bias among contributing studies.	Pages 6-13 & Tables 1-2
	20b	Present results of all statistical syntheses conducted. If meta-analysis was done, present for each the summary estimate and its precision (e.g. confidence/credible interval) and measures of statistical heterogeneity. If comparing groups, describe the direction of the effect.	NA
	20c	Present results of all investigations of possible causes of heterogeneity among study results.	NA
	20d	Present results of all sensitivity analyses conducted to assess the robustness of the synthesized results.	NA
**Reporting biases**	21	Present assessments of risk of bias due to missing results (arising from reporting biases) for each synthesis assessed.	Page 5 & Supplementary Table 2
**Certainty of evidence**	22	Present assessments of certainty (or confidence) in the body of evidence for each outcome assessed.	Table 1
**DISCUSSION**			
**Discussion**	23a	Provide a general interpretation of the results in the context of other evidence.	Page 13
	23b	Discuss any limitations of the evidence included in the review.	Page 14-16
	23c	Discuss any limitations of the review processes used.	Page 16
	23d	Discuss implications of the results for practice, policy, and future research.	Page 17-18
**OTHER INFORMATION**			
**Registration and protocol**	24a	Provide registration information for the review, including register name and registration number, or state that the review was not registered.	NA
	24b	Indicate where the review protocol can be accessed, or state that a protocol was not prepared.	NA

Topic	No.	Item	Location where item is reported
	24c	Describe and explain any amendments to information provided at registration or in the protocol.	NA
**Support**	25	Describe sources of financial or non-financial support for the review, and the role of the funders or sponsors in the review.	Page 20
**Competing interests**	26	Declare any competing interests of review authors.	Page 20
**Availability of data, code and other materials**	27	Report which of the following are publicly available and where they can be found: template data collection forms; data extracted from included studies; data used for all analyses; analytic code; any other materials used in the review.	Table 1 & 2

PRIMSA Abstract Checklist

Topic	No.	Item	Reported?
**TITLE**			
**Title**	1	Identify the report as a systematic review.	Yes
**BACKGROUND**			
**Objectives**	2	Provide an explicit statement of the main objective(s) or question(s) the review addresses.	Yes
**METHODS**			
**Eligibility criteria**	3	Specify the inclusion and exclusion criteria for the review.	No
**Information sources**	4	Specify the information sources (e.g. databases, registers) used to identify studies and the date when each was last searched.	No
**Risk of bias**	5	Specify the methods used to assess risk of bias in the included studies.	No
**Synthesis of results**	6	Specify the methods used to present and synthesize results.	No
**RESULTS**			
**Included studies**	7	Give the total number of included studies and participants and summarise relevant characteristics of studies.	Yes
**Synthesis of results**	8	Present results for main outcomes, preferably indicating the number of included studies and participants for each. If meta-analysis was done, report the summary estimate and confidence/credible interval. If comparing groups, indicate the direction of the effect (i.e. which group is favoured).	Yes
**DISCUSSION**			
**Limitations of evidence**	9	Provide a brief summary of the limitations of the evidence included in the review (e.g. study risk of bias, inconsistency and imprecision).	Yes
**Interpretation**	10	Provide a general interpretation of the results and important implications.	Yes
**OTHER**			
**Funding**	11	Specify the primary source of funding for the review.	No

Topic	No.	Item	Reported?
**Registration**	12	Provide the register name and registration number.	No

From: Page MJ, McKenzie JE, Bossuyt PM, Boutron I, Hoffmann TC, Mulrow CD, et al. The PRISMA 2020 statement: an updated guideline for reporting systematic reviews. MetaArXiv. 2020, September 14. DOI: 10.31222/osf.io/v7gm2. For more information, visit: www.prisma-statement.org
